# Bizarreness and Emotion Identification in Grete Stern Photomontages: Gender and Age Disparities

**DOI:** 10.3389/fpsyg.2017.00414

**Published:** 2017-03-22

**Authors:** Alejandra Rosales-Lagarde, Claudia Isabel Martínez-Alcalá, Patricia Pliego-Pastrana, Eva María Molina-Trinidad, José-Luis Díaz

**Affiliations:** ^1^Cátedras CONACyT, Consejo Nacional de Ciencia y TecnologíaMexico City, Mexico; ^2^Área Académica de Gerontología, Universidad Autónoma del Estado de HidalgoPachuca, Mexico; ^3^Área Académica de Medicina, Universidad Autónoma del Estado de HidalgoPachuca, Mexico; ^4^Departamento de Historia y Filosofía de la Medicina, Universidad Nacional Autónoma de MéxicoMexico City, Mexico

**Keywords:** bizarreness, emotion, art, Grete Stern, gender, age

## Abstract

Although the International Affective Picture System (IAPS) is used to evaluate emotions (valence, arousal, and dominance evoked by a large set of photographs), bizarre images in works of art have not been assessed with the IAPS procedures. Understood here as strange, non-sense, and absurd mental contents or expressions accompanied by surprise and confusion emotions, bizarreness was assessed after healthy adult volunteers assigned this specified variable to 140 Grete Stern's photomontages overtly intended to illustrate strange, absurd, and non-sensical contents in dream reports. The images were presented to 21 Young Males (YM) and 30 Young Females (YF) who were instructed to use the IAPS Self-Assessment Manikin, along with an additional bizarre-to-normal scale, to evaluate their response to them. The valence and the bizarre-to-normal ratings showed a dissimilar pattern of distribution between genders. Ratings of scales were different, and a greater variation in scales occurred according to gender. When bizarreness was appraised, gender differences became more evident especially for YF, who rated half of the images as bizarre, and with a diminished feeling of control, while the neutral and normal images were deemed more pleased and controlled. Valence, bizarreness, and dominance formed a different component than arousal in both groups. Negative correlations between valence and dominance, and between valence and bizarreness were also found in both groups, plus a positive one for dominance and bizarreness in YF, along with curvilinear relationships among all scales. On a second experiment, 10 photomontages evaluated by YF as *bizarre* or as *normal* were administered to 18 Old Males (OM) and 28 Old Females (OF). OF's arousal showed less neutral evaluations than OM's. In OF the bizarre images evoked either more excitation or calmness than in OM. The distribution of the bizarre-to-normal scale was significantly different across the evaluations in YM, YF, OM, and OF. The use of this extended IAPS instrument to explore bizarreness and emotional variables in response to art images seems suitable and potentially valuable to characterize bizarre, absurd, or non-sensical mental states and their brain correlates.

## Introduction

Mental or expressive “bizarreness” is an important but complex and vague concept, difficult to define and measure. This alleged property of some mental processes and pictorial or verbal expressions has not been clearly identified and characterized (Cermolacce et al., 2010). Recently the term “bizarre delusions” in psychotic disorders has been eliminated in the DSM-V (Tandon et al., 2013; Manual of the American Psychiatric Association., 2014). Nevertheless, since multiple features and expressions, such as incongruities, contradictions, and paradoxes occurring in natural and human domains prevent subjects to engage in credible representations and appropriate actions, the concept of mental bizarreness still constitutes a relevant empirical and theoretical challenge.

Hall and Van de Castle (1966) specified the following words in dream reports to identify mental confusion: surprised, astonished, amazed, awestruck, mystified, puzzled, perplexed, estranged, bewildered, doubtful, conflicted, undecided, and uncertain. Domhoff (2007) define bizarreness as distorted settings, metamorphosed characters, or feelings of confusion and surprise resulting from unexpected events. Cermolacce et al. (2010) identify bizarreness in contrast with congruous ordinary experience as non-sense, incongruity, physical or logical impossibility, implausibility, and incomprehensibility. In a book about non-sense, Cappuccio and Froese (2014) emphasize that the disturbing feeling of the unfamiliar, strange or bizarre usually directs attention and self-monitoring functions toward the generation of action-oriented representations. In a chapter of this book, González (2014) further stipulates that a perceived non-sense defies the agent's rationality to transform the non-sensical into sense-making and meaningful experience.

Following the cognitive/affective conception of bizarreness derived from these approaches and previous evidence in the present investigation we tentatively define mental bizarreness in the following three-fold manner: “(1) perceptions of non-sense, incongruity, distortion and physical or logical impossibility, implausibility or incomprehensibility, (2) involving feelings of confusion, surprise and strangeness, that (3) are identified in contrast with habitual, congruous, logical and meaningful experiences.” In the present study we demonstrate that a first-person method extending the IAPS emotion system to evaluate bizarreness in photomontages crafted to depict dream scenes is a valuable tool to compare this otherwise elusive phenomenon between human genders and age groups, and to correlate it to emotional variables such as valence, arousal, and dominance.

Several instruments have been applied to measure incongruous, non-sensical, and bizarre mental states in psychopathology and cognitive psychology. The *Dissociative Experiences Scale* is a self-report assessment that evaluates absorption, depersonalization/de-realization, and amnesia (Bernstein and Putnam, 1986; Van Heugten-van der Kloet et al., 2014). The *Examination of Anomalous Self-Experience* (EASE) is a 57-item semi-structured interview focusing on self-affection, hyper-reflectivity, “disturbed” hold on the world, or confusion with others (Parnas et al., 2005; Sass, 2014; Sass and Byrom, 2015). In the classic Hall and Van de Castle (1966) analysis of dreams, the emotion of confusion is categorized apart from a group of distorted places, characters, creatures, and metamorphoses. Domhoff (2007) considers both of these categories as “bizarre.”

Allan Hobson's group has analyzed dream bizarreness as content incongruity, discontinuity, or uncertainty in three cognitive categories: (1) bizarreness of place, plot, object, character, time, and action, (2) bizarreness of thought, and (3) bizarreness of emotion (Williams et al., 1992; Merritt et al., 1994; Scarone et al., 2008; D'Agostino et al., 2010). While incongruity and discontinuity were found to be the most frequent, followed by uncertainty of thought (Williams et al., 1992; Scarone et al., 2008), uncertainty of plot and thought were difficult to distinguish (Williams et al., 1992). In a study of dream contents judged for bizarreness, Revonsuo and Salmivalli (1995) found that dream emotions had a lower rate of incongruity (11.8%) than animate objects (15.1%), persons (15.2%), objects (16.1%), events (23.2%), language (31%), and cognition (34.7%). Compared to similar waking episodes, “bizarre” experiences occurring during Rapid Eye Movement (REM) phases emerge in an involuntary manner, and occur more frequently, in contrast with Non REM dream mentation (Fosse, 2000; Fosse et al., 2001; Corsi-Cabrera et al., 2003). Moreover, scenes and items are not usually perceived as bizarre or non-sensical during the original dream experience, but judged as such during their wakeful recollection and narration (Díaz, 2015).

It has been difficult to distinguish “bizarreness” among dream reports of normal subjects and waking mentation of schizophrenics (Noreika et al., 2010) and major depressives (Cavallotti et al., 2014). “Bizarreness” understood as a wakeful-dreaming pathological state, has been induced by images evoking projective interpretations in order to measure psychotic mentation. Using this approach, Scarone et al. (2008) found that seven Thematic Apperception Test (TAT) pictures elicited a higher percentage of “bizarre” responses in schizophrenic compared to normal subjects. When the habitual knowledge of the world is tested, “bizarre” answers have been more frequently found in frontal-damaged patients vs. patients with lesions of posterior areas (Shallice and Evans, 1978; MacPherson et al., 2014). Bizarreness and emotion have been reported as decreased in patients with basal ganglia bilateral damage (Leu-Semenescu et al., 2013). Cognitive studies have employed deformed images or impossible sentences to originate bizarre feelings and judgments in healthy people. The “bizarreness effect” phenomenon obtained with these techniques refers to the fact that unexpected, distinctive or bizarre items, sentences and images are remembered more easily than common ones (Von Restorff, 1933; Hunt, 2006; Geraci et al., 2013; Gounden and Nicolas, 2015).

**Figure 1:**
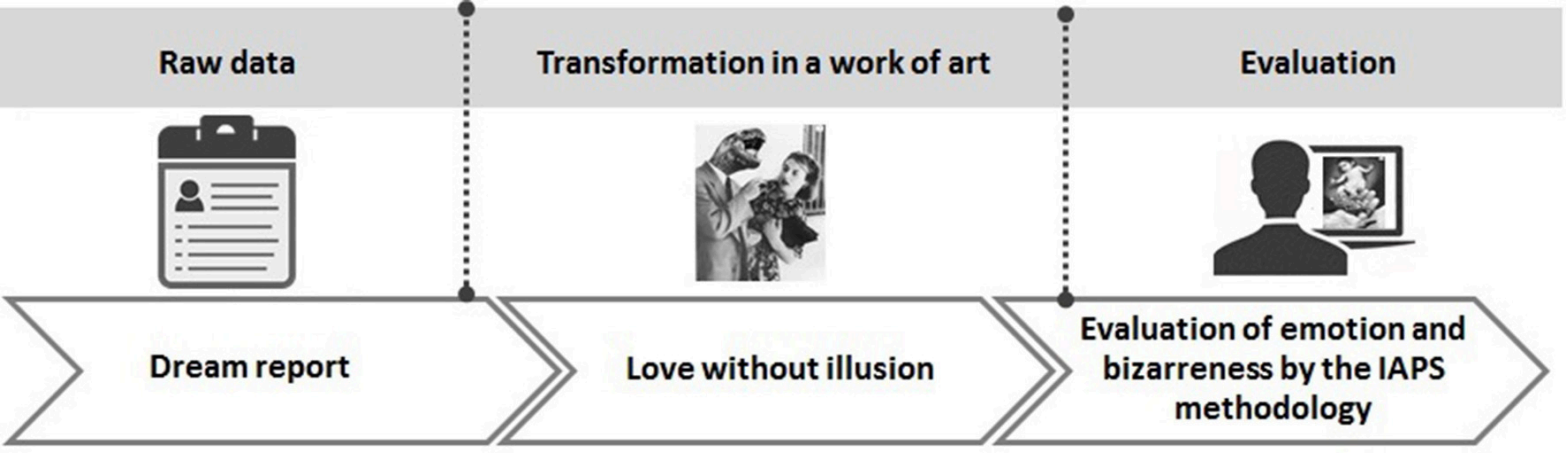
**Dream reports were interpreted by trained psychoanalysts and rendered into photomontages by Grete Stern and collaborators**. Next, the evaluation of bizarreness and emotions by young students and later by old adults was carried on. Photomontages from Stern et al. (2012) are reproduced with permission.

We propose now that Grete Stern photomontages inspired in dream reports can be used to specify and measure mental bizarreness. Grete Stern, a German artist, crafted 140 photomontages that were published between 1948 and 1951 in the weekly magazine *Idilio* of Argentina. The magazine requested female readers to submit written accounts of dreams. Salient scenes of the dream reports selected for psychoanalytic interpretation were illustrated by the artist (Stern et al., 2012). These photomontages usually depict non-sensical and absurd disproportions, fragmentations, and other logical and/or factual incongruities typical of dreams (Díaz, 2015). In order to evaluate not only emotion, but also bizarreness, in the present study we extend the methods employed by the International Affective Picture System (IAPS, Lang et al., 2005; Figure 1). The IAPS has been previously extended to measure compassion in both men and women (Mercadillo et al., 2007) and in the present experiments, one scale was added in which, in accord with the above definition, the IAPS manikin exhibited an expression of strangeness in contrast to a neutral gesture.

With the application of this novel instrument, we explored possible dissimilarities between genders since the frequency of distorted places or metamorphoses found in dream reports is about double in women vs. men (Hall and Van de Castle, 1966; Domhoff, 2007), and gender differences have been found in several emotions evaluated with the IAPS methodology (Bradley et al., 2001; Lang et al., 2005; Mercadillo et al., 2007; Silva, 2011), Moreover, we analyzed bizarreness in young and old people because dreams (Giambra, 1980), daydreaming activity (Grenier et al., 2005; Guénole et al., 2010), and the “bizarreness effect” (Smith, 2006) have been reported to decrease with age. In order to explore the bizarreness evoked by the selected graphic material in young and old men and women, intra and intergroup gender and age differences were studied in terms of frequencies, relationships, comparisons of means, and principal component analysis.

## Methods

### Experiment 1

#### Participants

The images were evaluated by 51 college students (21 Young Males, YM, and 30 Young Females, YF, 21.86 ± 2.64 years of age with no significant difference between genders) from the Gerontology program of the *Universidad Autónoma del Estado de Hidalgo* in the city of Pachuca, México. The experiment was part of these students' field practices. Students were told that their evaluations of the images had to be immediate, as the instructions of the IAPS demands, so no relation to an age-study bias seemed to occur. A letter of consent was read and signed by all subjects. This research was part of a larger project called “Design of tests to pre-diagnose and diagnose Old Adults of Hidalgo at the bio-psycho-techological areas” and received the approval of the research Ethics committee.

#### Images and task

**Figure 2:**
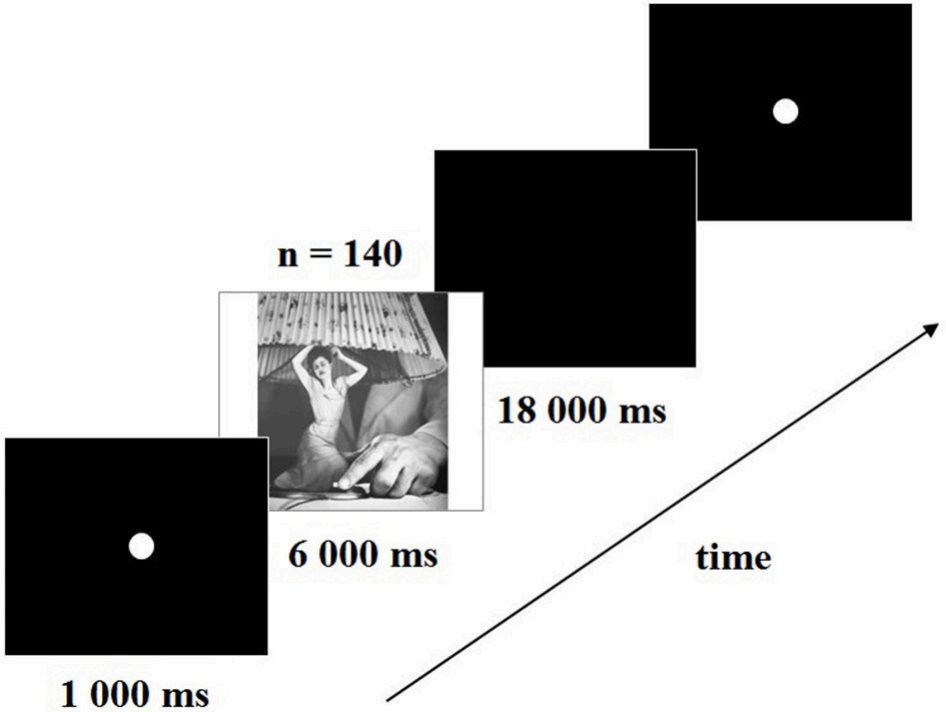
**Presentation timing of the task**. A Stern photomontage is preceded by a fixation point. After the image, a black screen appears and ratings using the modified Self-Assessment Manikin format are requested.

From the original set of Grete Stern's photomontages (Stern et al., 2012), some were substituted by later versions of the artist (i.e., “Love without illusion” an improved version of “Idilio 64”; “Made in England”; or “Paintbrush dreams” instead of “Idilio 101”) and others discarded because they were not fully discernable in the computer screen (i.e., “Extrañamiento”). A total of 140 Grete Stern photomontages (see Supplemental Material) were presented on the center of a 64 × 113 cm computer screen. Following a 1 s fixation point period, each image was presented during 6 s, and evaluated on four scales during an inter-trial black screen of 18 s (Figure 2).

#### Procedure

The traditional IAPS written instructions were administrated along with this phrase (translated from Spanish): “We are interested in how people respond to pictures that represent different events that can or cannot occur in life.” Since the IAPS allows for explanations of the instructions to assess emotions, the bizarreness-to-normal category stated: “You will see four sets of five figures, and you will use these figures to rate how you felt while viewing each picture. You will make four ratings for each picture that you observed. The manikins show four different kinds of feelings: Joy vs. Sad, Excited vs. Calm, Controlled vs. In-Control, and *Extrañado* vs. Normal.” The latter and added scale was explained in this way:

“The last of the scales is about the feeling of bizarreness, strangeness or perceiving an absurd (*extrañado, sorprendido o percibiendo algo absurdo*). In such events you will be putting an X on the figure on the left, like this (demonstrate with the manikin). If you felt completely normal, as having a familiar and common experience you will indicate it with an X on the figure on the right (demonstrate with the manikin). Note the figure on the left has a bizarre or surprised expression and that on the extreme right a neutral expression. If you did neither feel “*extrañado*” nor “normal,” put an X in the middle figure.”

**Figure 3:**
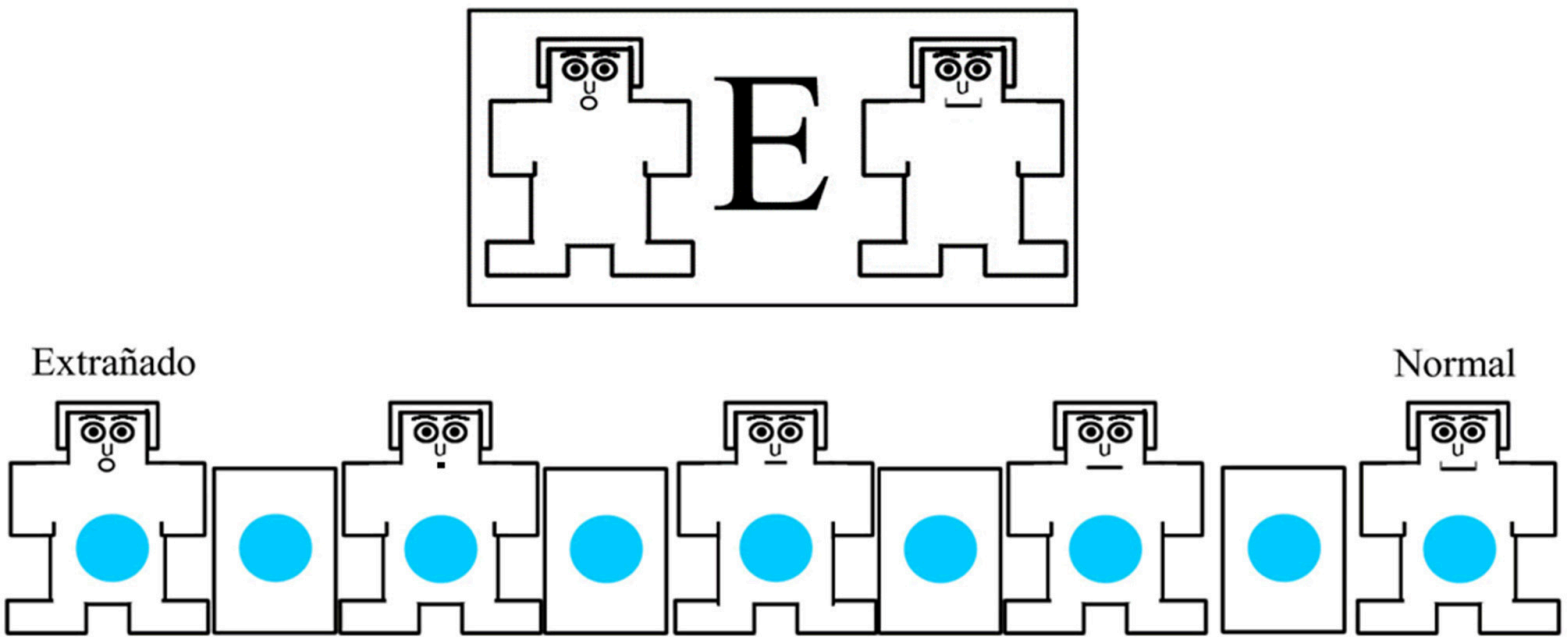
**Modified sheet format of the Self-Assessment Manikin of the International Affective Picture System for Young Adults with the additional bizarreness scale**. For the 140 Stern photomontages, 10 per sheet were rated for *Valencia* (Valence), *Alertamiento* (Arousal), *Dominancia* (Dominance), and *Extrañeza* (Bizarreness).

According to the original IAPS system, the subjects had to rate Valence (glad vs. sad), Arousal (excited vs. calmed), Dominance (being dominated vs. dominate) using the usual manikins, plus a rating of Bizarreness with the aid of an additional manikin. The introduced manikin had a round-open mouth to indicate “*extrañado*” (ratings 1, 2, 3, and 4 to indicate bizarreness) and another one with a neutral expression to indicate a usual or “normal” condition (ratings 6, 7, 8, or 9) while 5 meant neither one nor the other (Figure 3). A sheet contained the four scales for 10 images. To balance the conditions of valence, arousal, dominance, and the bizarre scales, four orders of the manikins were placed on the format.

#### Statistical analysis

Means and standard deviations were obtained for each of the 140 pictures in the scales of valence, arousal, dominance, and bizarre-to-normal. Afterwards, percentages to visualize how the ratings were distributed, and chi-squares to compare frequency of choice between groups were used. To know which relationships were followed by the variables, linear correlations among the possible six combinations of variables from the 140 means across images and curvilinear regressions were tested. Since the data followed a normal distribution and in order to compare the means, ANOVAs with gender and emotional variables as factors were implemented. Tukey *post-hoc* tests for comparisons of means were then used. A PCA was done to reduce the number of variables. As the variable of interest, the bizarreness scale was used to discriminate images within each gender using dependent Student *t*-tests and later between gender groups with independent Student *t*-tests.

### Experiment 2

#### Participants

The sample of older people from the city of Pachuca and nearby locations (administrative university workers, grandparents, or acquaintances of participant students) was made of 28 Old Females (OF; 68.41 ± 8.19) and 18 Old Males (OM; 68.12 ± 7.45). There were no significant differences between the two groups concerning age, education, or results of the Mini-Mental State Examination (MMSE), Geriatric Depression Scale (GDS), Short Anxiety Screening Test (SATS), or Katz Daily Activities Scale in their Spanish versions (Ugalde, 2010; Table 1). Education ranged from 3 analphabet subjects to 15 years of education. Some of the subjects wore glasses or auditory devices.

**Table 1:** **Mean and standard deviation of age; education MMSE, Mini-Mental State Examination; GDS, Geriatric Depression Scale; SATS, Short Anxiety Screening Test and Katz scale for Old Aged subjects**.

	**Old males**	**Old females**	***t***	***p***
Age	68.12 (7.45)	68.41 (8.19)	1.35	0.90
Education (years)	7.5 (6.24)	6.7 (5.23)	0.88	0.81
MMSE	26.69 (4.01)	25.41 (3.79)	−0.38	0.35
GDS	5.47 (4.72)	4.83 (3.86)	0.38	0.64
SATS	19.55 (4.01)	20.83 (2.5)	−0.54	0.29
Katz	0.62 (1.66)	0.21 (0.54)	−0.45	0.32

*Level of significance set at p < 0.05*.

#### Images and task

**Figure 4:**
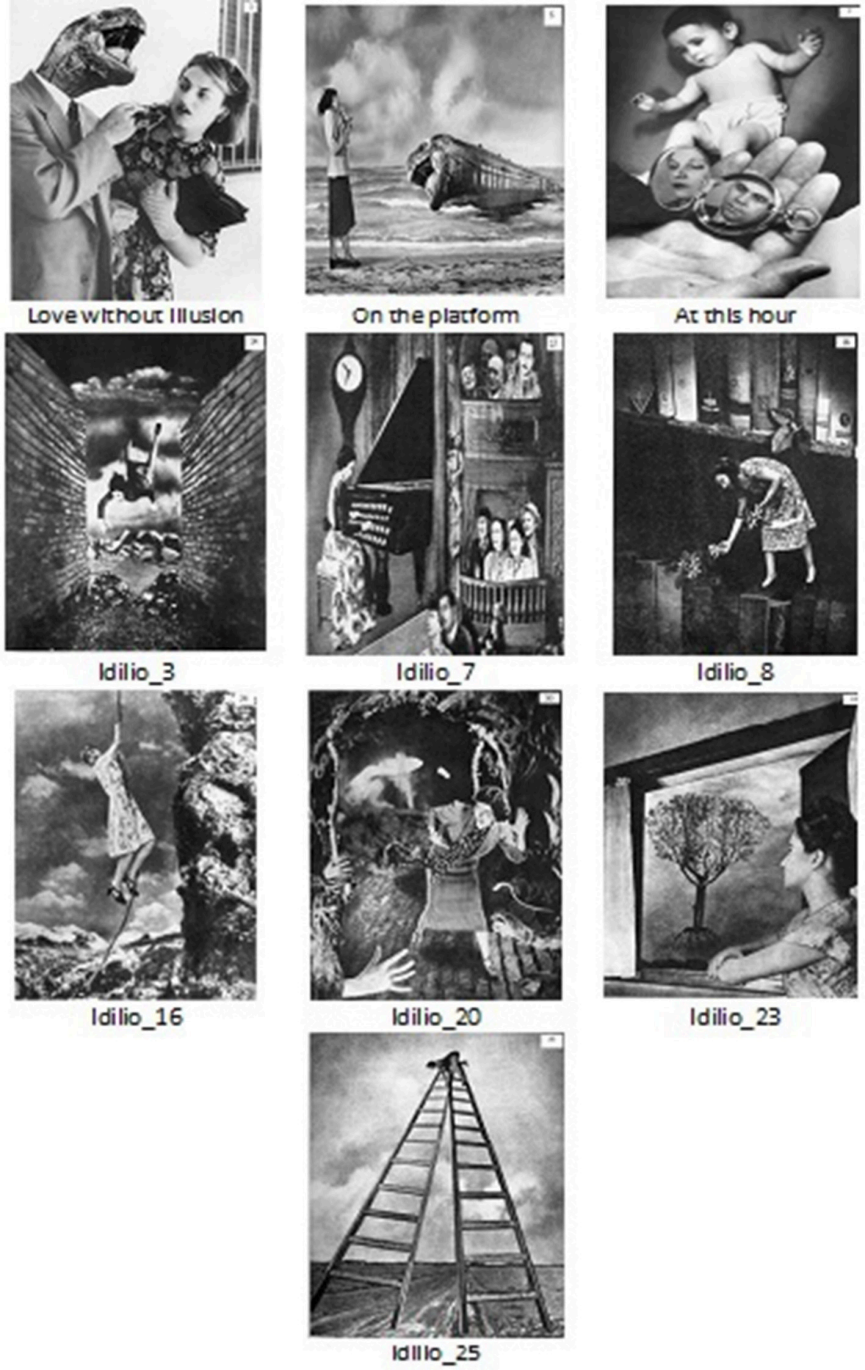
**Stern photomontages evaluated with the extreme values of 3 as “bizarre” or 7 as “normal” by most of the four groups**. Of the 10 photomontages rated by young and old subjects, “Idilio 16” was bizarre for young adults. Only “Idilio 20” was bizarre for old and young women. Old male subjects rated neither of them as extremely bizarre or normal. Photomontages from Stern et al. (2012) are reproduced with permission.

Black and white Stern photomontages were exhibited on a computer screen. Old Adults (OA) viewed similar manikins as in Figure 3 with the four scales. In contrast to Young Adults (YA), OA evaluated one image per sheet of paper, but the scales were enlarged in order to facilitate visualization and evaluation. Depending on the individual capacity for execution or proneness to become tired, 10–40 pictures were shown. These were selected from the study with the college students with mean evaluations of 2, 3, or 4 (bizarre) or 6 or 7 (normal). The values for the first 10 images are presented. The photomontages judged as bizarre by YF were “Love without illusion,” “On the platform,” “Idilio 3,” “Idilio 16,” “Idilio 20,” and “Idilio 25.” The “normal” images were “At this hour,” “Idilio 8,” and “Idilio 23” by the group of YM and “Idilio 7” by YF (Figure 4).

#### Procedure

The instruction manual and the format for evaluating IAPS were used, both modified to include the bizarreness scale. After instructions, each one of the Stern photomontages was presented on the center of the screen, but was not time restricted to avoid visual or speed difficulties. Two different sequences balanced the position to a particular series of images. To diminish possible effects of unfamiliar settings and techniques (Lupien et al., 2007), OA were evaluated at their homes and photomontages were shown on personal computers or laptops.

#### Statistical analysis

Since a differential evaluation was observed for each image considering valence, arousal, dominance, and the bizarreness scales, analyses were carried out according to the mean of each of the 10 images for the two groups and each variable and their frequencies were submitted to Pearson's chi-square tests. Later, chi-square tests were used for each scale to compare the distribution of responses along the four groups.

## Results

### Experiment 1

In Tables 2, 3, the mean ratings and standard deviations in YM and YF for “bizarre” and “normal” photomontages are shown. When submitted to chi-square tests, frequencies of valence $\chi_{\left(5\right)}^{2}$ = 20.94, *p* = 0.0003, and the bizarre to normal scale $\chi_{\left(5\right)}^{2}$ = 15, *p* = 0.01, were significant. Stern's images in YF evoked more extreme ratings on the valence and the bizarreness scale than YM. Thus, from the 140 images, YM rated none as extremely bizarre (*M* = 2) and 5.71% as moderately bizarre (*M* = 3), while YF rated 1.4% (*M* = 2) and 18.57% (*M* = 3). YM rated 13.57% as normal (*M* = 6) or moderately normal 1.42% (*M* = 7), while YF evaluated 15 and 4.2% as normal or moderately normal. When the means of 2, 3 and 4 for bizarreness were considered, the result for YM was 45.71%, while for YF was 50%. The figures for normality (6 or 7) were 15% for YM, and 19.28% for YF. “Idilio 16,” “Idilio 46,” and “Idilio 55” were considered bizarre by both sexes. Only “Idilio 124” showed normal ratings between genders. “Idilio 23,” a picture of a woman watching a flying tree from her window, was rated as *normal* by YM. Similarly “Idilio 7,” showing a woman playing a piano with typewriter keys was rated as *normal* by YM.

**Table 2:** **Means and standard deviations (in parentheses) of “bizarre” (mean of 2 or 3 in bold) and of “normal” photomontages (mean of 7 in plain text) rated by YM**.

**Young Males**					
**Name**	**Slide no**.	**Valence**	**Arousal**	**Dominance**	**Bizarreness**
“ATH”	7	3.94 (2.86)	6.75 (2.82)	5.69 (2.15)	7.50 (1.86)
“Idilio 1”	12	3.67 (2.20)	5.95 (2.64)	5.65 (1.84)	**3.86 (2.52)**
“Idilio 8”	18	3.75 (2.62)	6.69 (2.73)	5.27 (2.12)	7.19 (2.37)
“Idilio 16”	26	5.14 (2.50)	6.35 (2.48)	4.75 (2.38)	**3.67 (1.62)**
“Idilio 23”	33	5.00 (3.16)	6.53 (2.10)	5.67 (1.63)	7.13 (2.96)
“Idilio 27”	37	5.95 (2.16)	4.20 (1.85)	5.15 (2.35)	**3.86 (2.39)**
“Idilio 34”	44	5.10 (2.72)	6.38 (2.50)	6.29 (2.92)	7.33 (1.93)
“Idilio 36”	46	3.71 (2.51)	4.86 (2.33)	6.24 (2.21)	**3.90 (2.83)**
“Idilio 46”	55	4.67 (2.48)	4.86 (2.76)	5.24 (2.76)	**3.57 (2.11)**
“Idilio 55”	64	5.80 (2.28)	4.45 (2.24)	5.35 (2.37)	**3.85 (2.74)**
“Idilio 83”	88	6.10 (2.85)	4.37 (2.43)	4.40 (2.91)	**3.65 (2.56)**
“Idilio 121”	123	5.52 (1.12)	5.62 (1.88)	4.19 (1.99)	**3.86 (2.17)**
“Idilio 124”	126	3.43 (1.80)	5.19 (2.80)	5.90 (1.55)	7.24 (2.28)

“ATH”, “At this hour.”

**Table 3:** **Same as Table **2** rated by YF**.

**Young Females**					
**Name**	**Slide no**.	**Valence**	**Arousal**	**Dominancee**	**Bizarrenesss**
“LWI”	1	4.43 (1.70)	6.30 (2.39)	4.41 (2.40)	**3.30 (2.74)**
“OTP”	6	5.55 (2.13)	4.43 (2.43)	4.30 (2.42)	**3.27 (2.56)**
“Idilio 3”	14	6.13 (2.26)	4.55 (2.47)	4.69 (2.32)	**3.14 (2.56)**
“Idilio 7”	17	3.41 (2.47)	7.41 (2.35)	6.03 (2.11)	7.13 (2.10)
“Idilio 16”	26	5.24 (2.50)	5.60 (3.11)	4.41 (2.32)	**3.79 (2.48)**
“Idilio 20”	30	5.62 (2.27)	5.03 (2.34)	4.28 (2.49)	**3.66 (2.36)**
“Idilio 25”	35	5.14 (2.26)	4.69 (2.55)	5.66 (2.51)	**3.79 (3.42)**
“Idilio 35”	45	6.23 (2.34)	4.93 (2.26)	3.90 (2.58)	**2.87 (2.87)**
“Idilio 45”	54	5.37 (2.63)	4.93 (2.75)	5.23 (2.22)	**3.67 (3.03)**
“Idilio 46”	55	6.70 (2.37)	4.86 (2.61)	3.97 (2.41)	**3.20 (2.75)**
“Idilio 50”	59	5.83 (2.38)	4.93 (2.38)	4.83 (2.00)	**3.73 (2.49)**
“Idilio 51”	60	7.23 (2.19)	4.50 (2.42)	4.00 (2.60)	**3.53 (2.83)**
“Idilio 55”	64	6.07 (2.02)	4.83 (2.68)	3.77 (2.22)	**3.30 (2.48)**
“Idilio 61”	69	5.07 (2.49)	5.07 (2.49)	3.93 (2.66)	**3.87 (3.15)**
“Idilio 67”	73	6.77 (1.81)	5.20 (2.37)	2.97 (2.11)	**2.90 (2.50)**
“Idilio 68”	74	5.33 (2.35)	6.03 (2.50)	4.30 (2.02)	**3.80 (2.91)**
“Idilio 71”	77	5.73 (2.60)	4.73 (2.50)	5.07 (2.60)	**3.67 (3.03)**
“Idilio 73”	79	3.57 (2.97)	5.20 (3.25)	4.33 (2.01)	7.37 (2.58)
“Idilio 74”	80	5.90 (2.38)	5.23 (2.28)	4.73 (2.16)	**3.67 (3.08)**
“Idilio 84”	89	6.67 (1.97)	5.00 (2.46)	4.17 (2.76)	**3.90 (2.76)**
“Idilio 86”	91	5.03 (1.63)	4.93 (2.30)	5.13 (2.30)	**3.33 (3.07)**
“Idilio 88”	93	7.67 (1.69)	6.33 (2.75)	4.20 (1.86)	**3.13 (2.57)**
“Idilio 89”	94	5.73 (1.70)	4.73 (2.66)	4.70 (2.42)	**3.77 (2.78)**
“Idilio 92”	96	6.30 (2.02)	4.80 (2.75)	3.87 (2.76)	**3.77 (2.92)**
“Idilio 97”	101	2.87 (2.34)	6.87 (2.83)	6.07 (2.33)	7.07 (2.80)
“Idilio 99”	103	4.33 (1.92)	4.93 (2.32)	4.40 (1.98)	**3.87 (3.14)**
“Idilio 100”	104	2.87 (2.52)	5.07 (3.22)	5.37 (1.79)	7.30 (2.51)
“Idilio 105”	108	5.47 (2.15)	5.60 (2.24)	3.73 (2.20)	**3.97 (2.93)**
“Idilio 115”	117	5.63 (2.50)	5.13 (2.67)	4.53 (2.15)	**3.13 (2.67)**
“Idilio 121”	123	5.97 (2.14)	5.30 (2.67)	4.21 (2.13)	**3.07 (2.80)**
“Idilio 124”	126	3.40 (1.71)	5.73 (2.85)	5.87 (2.34)	7.83 (1.70)
“Idilio 128”	130	2.77 (2.25)	5.47 (2.96)	5.83 (1.82)	7.27 (2.39)

“LWI”, “Love Without Illusion”; “OTP”, “On The Platform.”

**Figure 5:**
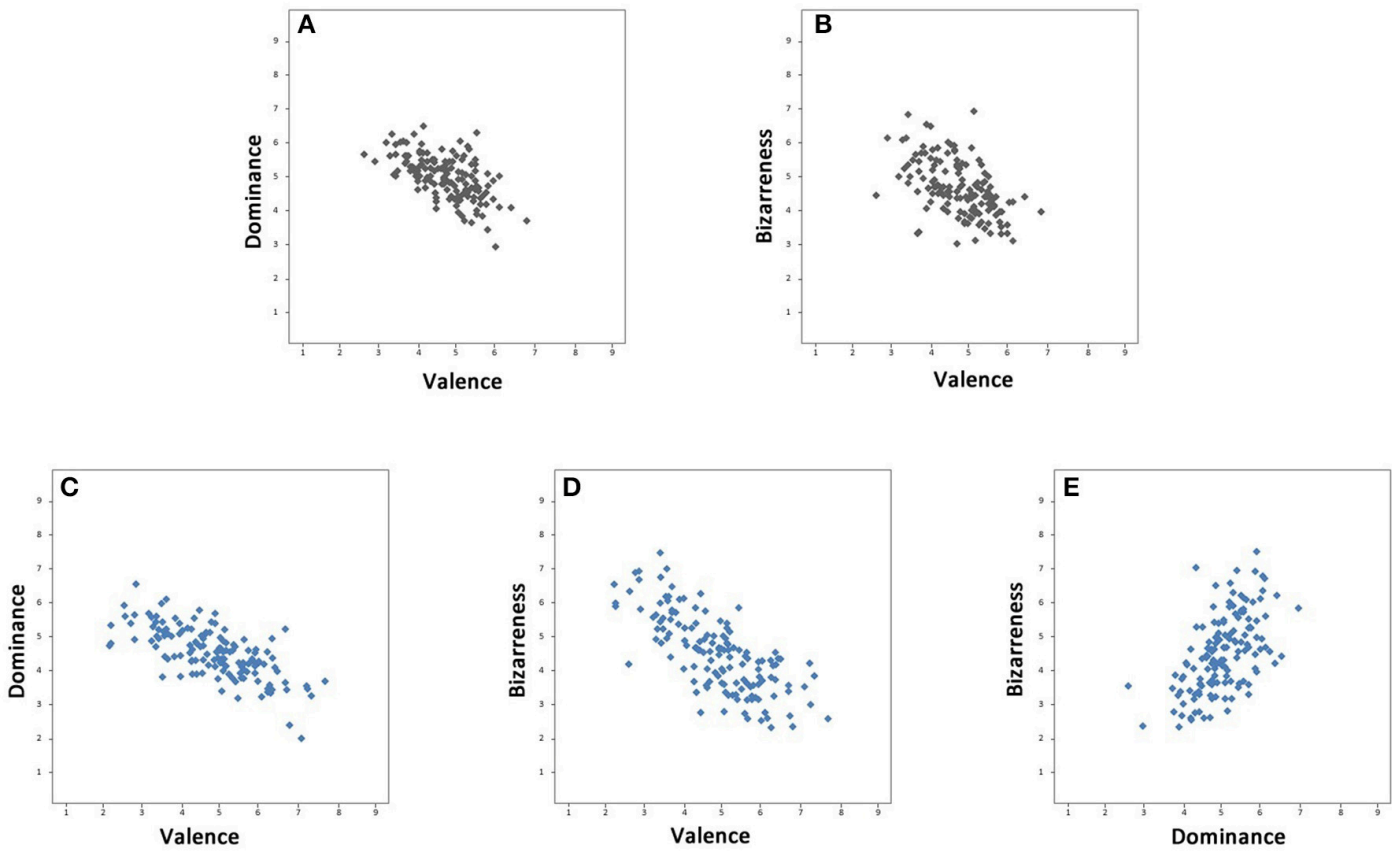
**Negative correlations in Young Males are shown in black between (A)** valence and dominance and **(B)** valence and bizarreness. The same correlations for Young Females **(C,D)**, and a positive one for dominance and bizarreness **(E)**.

Considering the six combinations of the four variables and the means of subjects for each one of the 140 photomontages, significant negative correlations were found between valence and dominance; and valence and the bizarreness scale for both sexes; for YM [*r*_(138)_ = –0.59, *p* < 0.001; *r*_(138)_ = −0.51, *p* < 0.001, respectively]; for YF [*r*_(138)_ = –0.69, *p* < 0.001; *r*_(138)_ = −0.73, *p* < 0.01, respectively], and YF associated positively dominance and the bizarreness scale, *r*_(138)_ = 0.56, *p* < 0.001. Figure 5 shows the scatter plots of the significant correlation values for YM and YF.

Quadratic regression analyses resulted in further evidence for associations of valence and dominance, valence and the bizarre scale, arousal and the bizarreness scale and dominance and the bizarreness scale in YM. All variable relationships were significant for YF and are presented in Table 4.

**Table 4:** **Significant quadratic regression analyses for emotion and the bizarreness scale by images in Young Males and Young Females**.

	**F**	**df**	**R^2^**	**p**
**YOUNG MALES**				
V-D	36.25	2, 137	0.35	<**0.001**
V-B	24.51	2, 137	0.26	<**0.001**
A-B	12.94	2, 137	0.15	<**0.001**
D-B	17.19	2, 137	0.20	<**0.001**
**YOUNG FEMALES**				
V-A	4.33	2, 137	0.07	<**0.001**
V-D	64.73	2, 137	0.48	<**0.001**
V-B	82.79	2, 137	0.54	<**0.001**
A-D	8.74	2, 137	0.11	<**0.001**
A-B	13.82	2, 137	0.16	<**0.001**
D-B	32.05	2, 137	0.31	<**0.001**

*Data are based on the mean score for each picture (n = 140)*.

*Valence (V), Arousal (A), Dominance (D), and Bizarre (B) scales. Numbers in bold indicate significant probability levels*.

**Figure 6:**
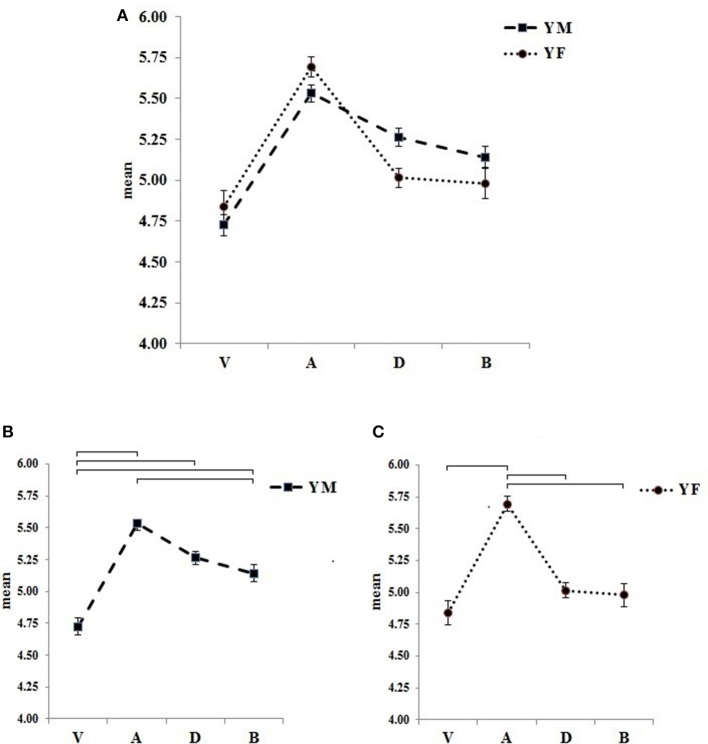
**Means and standard errors of the four scales: Valence (V), Arousal (A), Dominance (D), and the Bizarreness scale (B) according to gender for the 140 Stern photomontages**. YM (Young Males) and YF (Young Females) demonstrated an interaction between scales and gender **(A)**. In both YM (**B**, left) and YF (**C**, right) V-A and A-B differed. In YM, V-D and V-B; in YF, A-D. Significant results (*p* < 0.05) are indicated in brackets according to Tukey tests.

A mixed ANOVA (2 × 4) was employed to test the effects of gender, scales, and to compare whether there was an interaction between gender and scales. Although the evaluation of young men and women was not different [*F*_(1, 278)_ = 0.67, *p* = 0.58)], scales [*F*_(3, 834)_ = 43.20, *p* < 0.001)] and their interaction [*F*_(3, 834)_ = 3.76, *p* < 0.01] reached the probability threshold. Both sexes showed a peak in arousal at the middle of the scale in the neutral location (YM: *M* = 5.53, *SD* = 0.62; YF: *M* = 5.69, *SD* = 0.73), while valence and the bizarreness scales had the lower values (valence: YM: *M* = 4.72, SD = 0.77; YF: *M* = 4.84, *SD* = 1.15; the bizarreness scale: YM: *M* = 5.14, *SD* = 0.78; YF: *M* = 4.97, *SD* = 1.08). Tukey *post-hoc* tests revealed differences within genders, for valence and arousal; and arousal and the bizarreness scale varied significantly. These results confirm there is a specific effect of gender according to the evaluations: both YM and YF considered Stern photomontages to evoke joy, with a trend in YF to rate them as bizarre, while both groups indicated a neutral evaluation of arousal and dominance. Interestingly, arousal and dominance for YM were similar, a result not observed for YF (Figure 6).

In order to reduce variables, responses of both genders for the images were subjected to a Principal Component Analysis (PCA). Two components that explained 71.18% of the total variance PCA were identified. The first component explained 53.4% of the variance and was formed by valence, dominance, and the bizarreness scale. The second component explained 17.77% of the variance and was integrated by arousal (Table 5). According to the previous results, both genders differed among their variables from the arousal scale.

**Table 5:** **Principal Component Analysis of Grete Stern photomontages in both genders**.

**Eigenvectors Adjectives**		**1st Valence, Dominance, Bizarre**	**2nd Arousal**
**YOUNG MALES**			
*Alegre-triste*	(joy-sad)	−**0.88**	0.07
*Excitado-tranquilo*	(excited-calm)	0.10	**0.86**
*Ser dominado-dominar*	(be dominated-dominate)	**0.74**	0.05
*Extrañado-normal*	(bizarre-normal)	**0.68**	0.43
**YOUNG FEMALES**			
*Alegre-triste*	(joy-sad)	−**0.90**	−0.14
*Excitado-tranquilo*	(excited-calm)	0.14	**0.87**
*Ser dominado-dominar*	(be dominated-dominate)	**0.72**	0.31
*Extrañado-normal*	(bizarre-normal)	**0.80**	0.31
Eigenvalues		3.78	1.91
% of variance explained		53.4%	17.77%

*Varimax rotated values from Principal Component Analysis. Factor loadings higher than 0.5 are in bold. Eigenvalues higher than 1 and percentage of the total variance explained by eigenvectors are shown. Emotion adjectives were provided to subjects in Spanish and the English equivalents are shown in parenthesis*.

**Figure 7:**
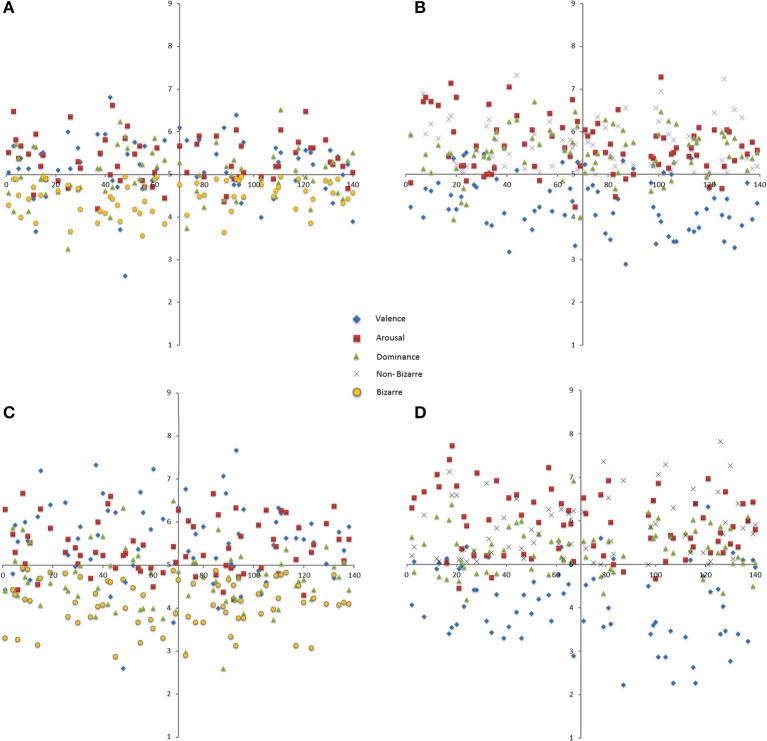
**Means of Stern images scatter plots split according to bizarreness (left) and to neutral-to-normal (right) evaluations in Young Males (A,B)** and Young Females **(C,D)**.

Furthermore, since the mean values of Stern images at the bizarre scale ranged from 2 to 7, and there was an interaction between gender and scales, pictures were split in two groups considering bizarre images as one group, and normal or neutral as a non-bizarre group of images. Figure 7A shows the scatter plots only for bizarre images in YM (*M* = 2, 3, and 4) and for every mean of valence, arousal, dominance, and the bizarreness scale (i.e., 64 bizarre images × 4 variables in YM = 256 means). In Figure 7C, the respective YF scatter plot is presented (70 bizarre images × 4 variables = 280 means). Means of 5, 6, and 7 (normality and neutrality; Figures 7B,D) for each gender group and each variable (76 normal or neutral images × 4 = 304 means for YM; 70 normal or neutral images × 4 = 280 means for YF) are found.

**Figure 8:**
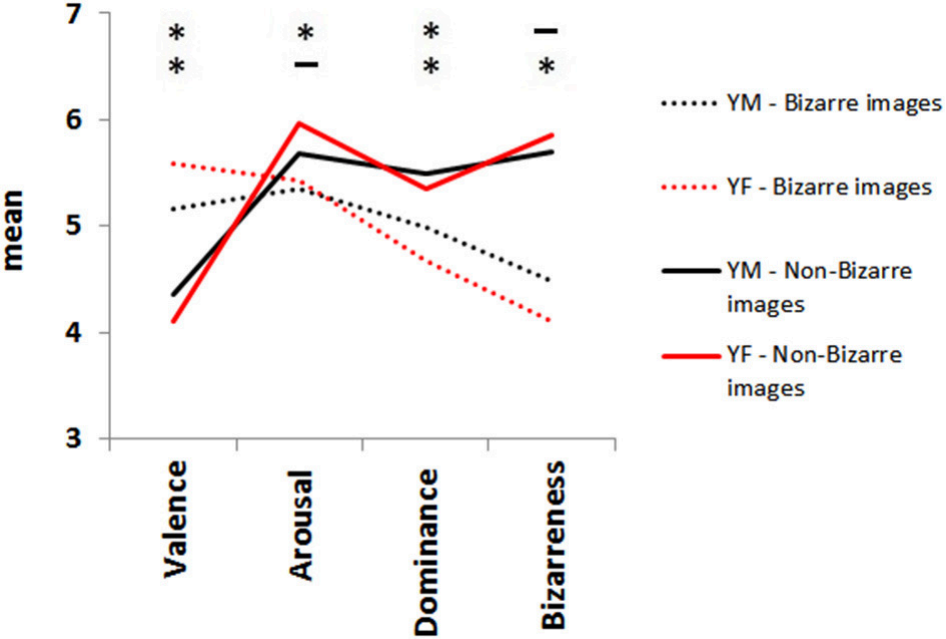
**Means of the data of the scatterplots in Figure **7****. Mean ratings in Young Males (in black) and Young Females (in red) separated Stern images into: bizarre (dotted lines), and neutral and normal images (continuous lines). Asterisks indicate significant differences between means. Level of significance set at *p* < 0.05.

There were significant differences in Student *t*-tests for every couple of variables according to gender. Valence of the bizarre images, *t*_(132)_ = 2.96, *p* = 0.003; valence of the normal and neutral images, *t*_(144)_ = 2.02, *p* = 0.04; arousal of the normal and neutral images, *t*_(144)_ = 2.42, *p* = 0.01; dominance of the bizarre images, *t*_(132)_ = 2.78, *p* = 0.006; dominance of the normal and neutral images, *t*_(144)_ = 3.83, *p* < 0.001; and the bizarreness scale of bizarre images, *t*_(132)_ = 4.6, *p* < 0.001, with the exception of arousal for the bizarre images [*t*_(132)_ = 0.79, *p* = 0.43] and the bizarreness scale for the normal and neutral images [*t*_(144)_ = 1.49, *p* = 0.14). As previously observed, higher scores for arousal ranging at the neutral score were also visualized, especially for non-bizarre images. As the continuous line shows, neutral and normal images are more happily rated (YM: *M* = 4.35, *SD* = 0.64; YF: *M* = 4.10, *SD* = 0.88) than those perceived as bizarre of the dotted lines (YM: *M* = 5.17, *SD* = 0.69; YF: *M* = 5.58, *SD* = 0.91), a more evident result for YF. Also, YF are more dominated by bizarre images than YM (YM: *M* = 4.98, *SD* = 0.59 vs. YF, *M* = 4.67, *SD* = 0.69). Bizarre images for YM were less bizarre (*M* = 4.48, *SD* = 0.38) than for YF (*M* = 4.10, *SD* = 0.55) than non-bizarre ones. Black lines (YM) come closer to the neutral rating of 5 than YF in red lines (Figure 8).

### Experiment 2

From the Pearson's chi-square tests for the 10 images in the OA groups, arousal was significant (χ^2^ = 19.95, *df* = 3, *p* < 0.001). While OM followed a normal distribution, choosing more neutral and calmer evaluations, OF followed an “L”- like distribution. Only “Idilio 3,” a woman falling, produced alertness in OM. “Love without illusion,” “At this hour,” “Idilio 7,” “Idilio 8,” and “Idilio 23” frequencies evoked greater calmness in OM. Although the bizarre scale was not differently distributed by sexes in old adults, of the 10 photomontages rated by young and old male and female subjects, considering extreme values (2, 3 or 7, 8), “Idilio 16” was bizarre for YA. Only “Idilio 20” was bizarre for OF and YF. OM subjects rated neither of them on the extremes as bizarre or normal, but they rated “Idilio 3” and “Idilio 16” with a mean of 4; and the other seven images with a mean of 5. “On the platform” was rated with the mean of 6. OF rated three of them with the mean of 4 (“Love without illusion,” “Idilio 3,” and “Idilio 25”); “At this hour,” “Idilio 7,” and “Idilio 23” with the mean of 6, and the other 2 pictures as more bizarre. “Idilio 8” and “Idilio 16” were rated as neutral. When a Pearson's chi-square test for the four groups of YM, YF, OM, and OF was done for the 10 images, the bizarre distributions were different across the groups (χ^2^ = 3.85, *df* = 12, *p* = 0.01), showing a similar pattern for YM and OF, more bizarre values for YF and more neutral ratings for OM (YM: 10, 30, 20, 40, and 0%; YF: 60, 10, 0, 20, 10%; OM: 0, 20, 70, 10, and 0%; OF: 20, 30, 20, 30, and 0%, for ratings of 3, 4, 5, 6, and 7, respectively). In Table 6, means and standard deviations of the most bizarre and normal images are shown for OA and YA.

**Table 6:** **Mean and Standard Deviations in parenthesis of “bizarre” (bold) and “normal” images (plain text) evaluated with the extreme values of 3 as “bizarre” or 7 as “normal” by the four groups**.

**Group**	**Name**	**Valence**	**Arousal**	**Dominance**	**Bizarreness**
**OLD MALES**					
**YOUNG MALES**					
	“Idilio 16”	5.14 (2.50)	6.35 (2.48)	4.75 (2.38)	**3.67 (1.62)**
**OLD FEMALES**					
	OTP	5.29 (2.42)	5.25 (2.61)	4.79 (2.45)	**3.64 (2.88)**
	“Idilio 20”	6.04 (2.86)	4.46 (3.08)	3.69 (2.40)	**3.24 (2.85)**
**YOUNG FEMALES**					
	“LWI”	4.43 (1.70)	6.30 (2.39)	4.41 (2.40)	**3.30 (2.74)**
	“OTP”	5.55 (2.13)	4.43 (2.43)	4.30 (2.42)	**3.27 (2.56)**
	“Idilio 3”	6.13 (2.26)	4.55 (2.47)	4.69 (2.32)	**3.14 (2.56)**
	“Idilio 7”	3.41 (2.47)	7.41 (2.35)	6.03 (2.11)	7.13 (2.10)
	“Idilio 16”	5.24 (2.50)	5.60 (3.11)	4.41 (2.32)	**3.79 (2.48)**
	“Idilio 20”	5.62 (2.27)	5.03 (2.34)	4.28 (2.49)	**3.66 (2.36)**
	“Idilio 25”	5.14 (2.26)	4.69 (2.55)	5.66 (2.51)	**3.79 (3.42)**

“LWI”, “Love Without Illusion”; “OTP”, “On The Platform.”

## Discussion

The present study was designed to assess emotion and bizarreness in response to Grete Stern's dream representations in photomontages by the application of the IAPS system extended to include bizarreness. Such first-person rating of mental states defined as strange, non-sensical, and absurd showed that it is possible to measure and standardize bizarreness originated from the inspection of pictorial stimuli. The overall results indicate that the experience of bizarreness encompasses both cognitive and emotional elements giving support to the first two components of our initial definition. Furthermore, the differential statistical profile of the images selected as bizarre and those chosen as normal or habitual reinforce the third part of our definition asserting that the stipulated array of cognitive and emotional characteristics of bizarreness stands in opposition to normal, habitual, and congruous expressions and experiences.

The distribution of the evaluation of images differed between young males and females in valence and bizarreness. When images were either separated or considered as a whole, YF registered more extreme ratings than YM in terms of joy, sadness, and bizarreness. The same applied to OF who rated images more at the extremes than OM. Males provide more neutral ratings, and this is more evident in OM. OF showed a different evaluation pattern than OM in the arousal scales, tending to be more responsive to the stimuli, reporting more excitement for some pictures and more calmness for others. The positive associations of dominance and bizarreness, and the negative associations of valence with dominance, and of valence and bizarreness summarize the mental effects of these images in YA. Calmer emotions and neutral ratings in the arousal scale seem to be evoked in YA. The evaluations of Stern photomontages follow a quadratic relationship similar to the boomerang-shaped distribution found for images of the IAPS (Lang et al., 1998). After the separation of photomontages by the bizarreness scale, the images split according to bizarreness. The more bizarre ones were deemed neutral and sadder and evoked a feeling of being controlled, while half of other images, rated as neutral and normal, were happier and evoked more dominance, especially in YF.

Both the gender and age effects found with the present instrument are consistent with the reports of higher frequency of dream bizarreness in YF (Hall and Van de Castle, 1966; Domhoff, 2007) and with the evidence obtained using the IAPS system that while men are more activated by erotica, women respond with greater defensive reactivity to aversive pictures (Bradley et al., 2001; Silva, 2011). It has also been found that the encoding of selected art stimuli has a gender and age effect, suggesting an attenuation of distinctiveness in OM (Smith, 2006). Old people seem to be less reactive and sensitive to anomaly, and spend less effort in attempting to resolve violations of expectancy.

Domhoff (2007) reported that bizarreness occurs in about half of the dreams reports if sudden changes, juxtapositions, uncertainty, confusion, and distension are considered in the evaluation. Using a similar definition, our results indicate that approximately half of Stern's images are evaluated as bizarre. Revonsuo and Salmivalli (1995) compared waking bizarreness to dreaming bizarreness and found the former was an adequate baseline of dream mentation, a hypothesis that could be tested using Stern's photomontages in future studies.

The present first-person method differs from written dream reports assessed by trained judges and constitutes an easier way, albeit less specific, to measure bizarreness. Certainly, formal analysis of written reports considers more information, but is less reliable than content analysis (Voss et al., 2011), and also requires independent judge agreement (Hall and Van de Castle, 1966). Alternatively, the IAPS method allows the control of the selection of emotional stimuli and facilitates the comparison and replication of results across different studies (Lang et al., 2005).

A larger and wider sample of subjects should be used in order to achieve a better understanding of the effects of these and other purportedly bizarre images such as Fineman's (2012) photoshop presentations. The present procedure can be employed to study bizarre and emotional states in response to diverse graphic or other art-related pictures. Furthermore, the technique may advance the knowledge of non-sensical or bizarre mentation in both psychopathology (Lang et al., 1998; Scarone et al., 2008) and neuroscience (see De Gennaro et al., 2011; Fox et al., 2013; Benedetti et al., 2015).

## Ethics statements

The study was approved by the Direccion de Investigacion de la Universidad Autonoma del Estado de Hidalgo, Oficio ICSa. GER/CAT/024/2015, 23/09/2015. Number: UAEH-DI-ICSA-GE-CF-008. Also, the ethics committee has approved the study. Bizarreness and Emotional Evaluations were carried out with the grandparents and friends of the students and did not imply any danger to them.

## Author contributions

JD contributed most to the idea about identifying bizarreness with Grete Stern images and rewrote the manuscript. AR implemented the overall evaluation, the methodology, and wrote the original article. CM and PP helped to gather the students to measure Grete Stern photomontages. CM also designed the sheet for the older adults and corrected the correlation figure. EM helped with the scatter plots of the results.

### Conflict of interest statement

The authors declare that the research was conducted in the absence of any commercial or financial relationships that could be construed as a potential conflict of interest.
