# Supplementary figures and images for: Bizarreness and Emotion Identification in Grete Stern Photomontages: Gender and Age Disparities
Source: Front Psychol. 2017 Mar 22;8:414. doi: 10.3389/fpsyg.2017.00414 (PMC5360721; doi:10.3389/fpsyg.2017.00414)

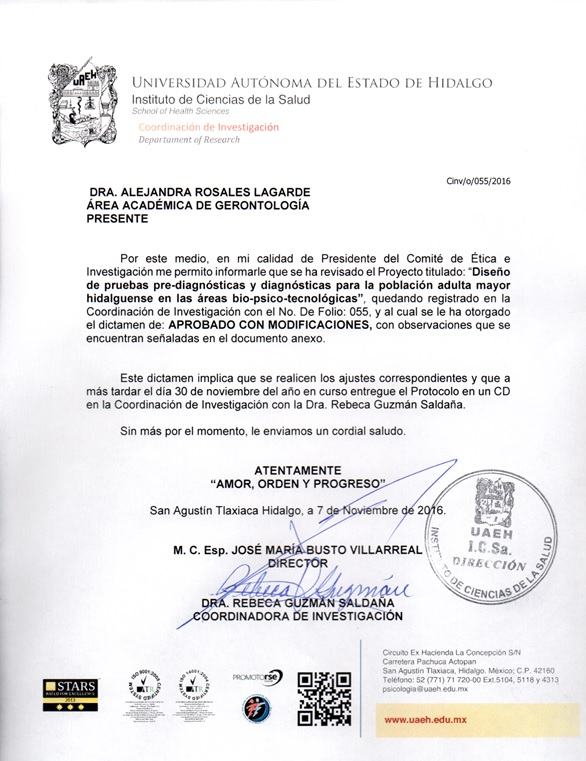

Supplement: Supplementary file 1 [file Image1.JPEG]
