# Supplementary material for: Bizarreness and Emotion Identification in Grete Stern Photomontages: Gender and Age Disparities
Source: Front Psychol. 2017 Mar 22;8:414. doi: 10.3389/fpsyg.2017.00414 (PMC5360721; doi:10.3389/fpsyg.2017.00414)
Supplement: Supplementary file 2 [file Table1.DOCX]

Supplementary Material

Bizarreness and emotion identification in Grete Stern photomontages: gender and age disparities

Alejandra Rosales-Lagarde*, Claudia I. Martínez-Alcalá, Patricia Pliego-Pastrana, Eva Molina-Trinidad, José-Luis Díaz

*** Correspondence:** alexiaro@rocketmail.com

# Table 1. Images reproduced with permission.

| 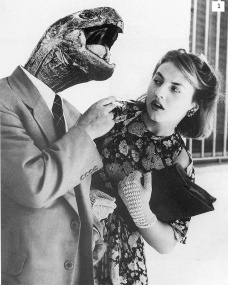 | 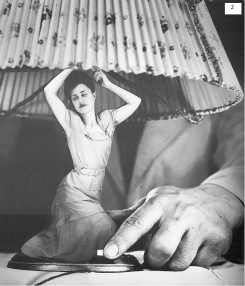 | 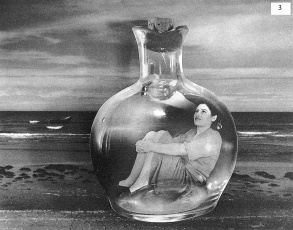 | 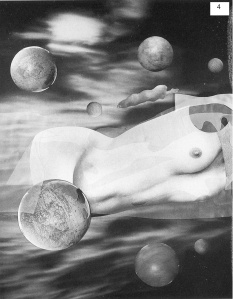 | 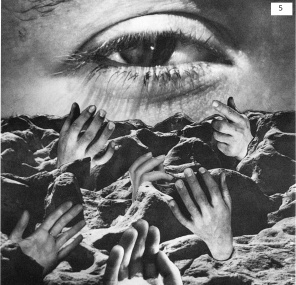 |
| --- | --- | --- | --- | --- |
| 1  “Amor sin illusion”  “Love without illusion” | 2  “Artículos eléctricos”  “Electric articles” | 3  “Botella de mar”  “Bottle of the sea” | 4  “Cuerpos Celestes”  “Celestial bodies” | 5  “El ojo eterno”  “The eternal eye” |
| 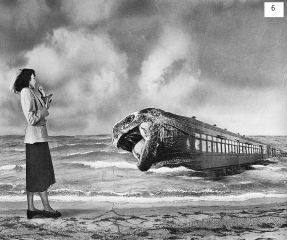 | 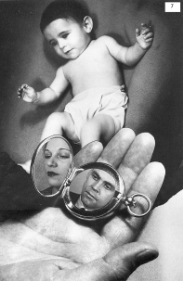 | 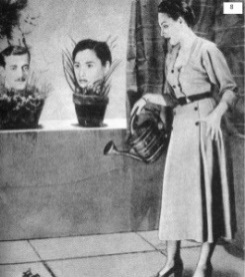 | 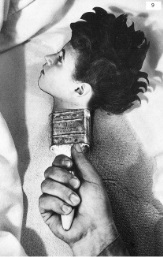 | 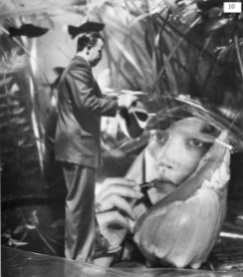 |
| 6  “En el Andén”  “On the platform” | 7  “En esta Hora”  “At this hour” | 8  “Los sueños de dominación”  “Domination dreams” | 9  “Made in England” | 10  “No destiñe con el agua”  “It does not fade with water” |
| 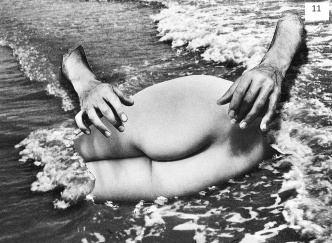 | 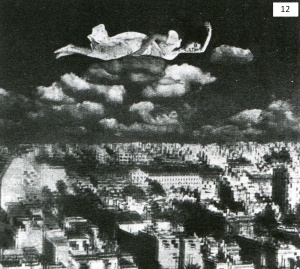 | 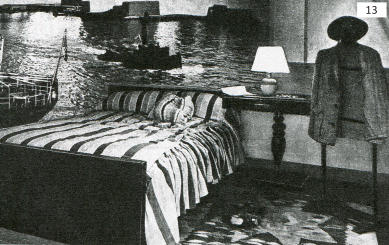 | 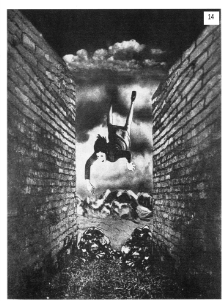 | 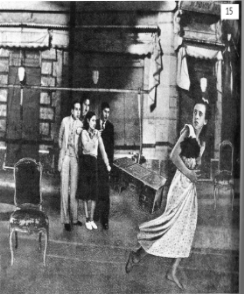 |
| 11  “Sirena de mar”  “Sea mermaid” | 12  “Idilio_1” | 13  “Idilio_2” | 14  “Idilio_3” | 15  “Idilio_4” |
| 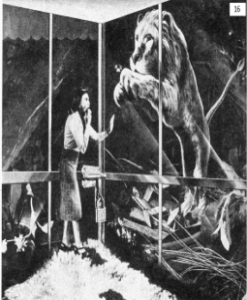 | 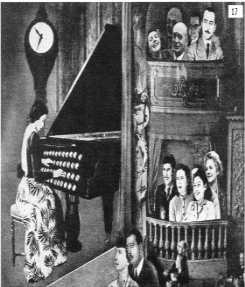 | 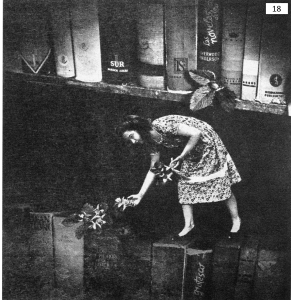 | 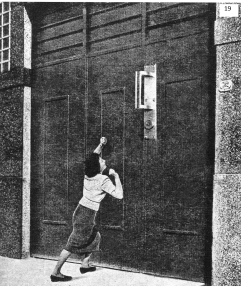 | 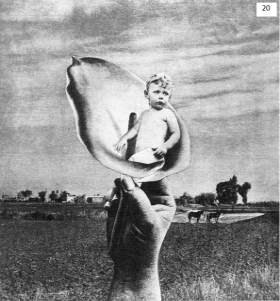 |
| 16  “Idilio_6” | 17  “Idilio_7” | 18  “Idilio_8” | 19  “Idilio_9” | 20  “Idilio_10” |
| 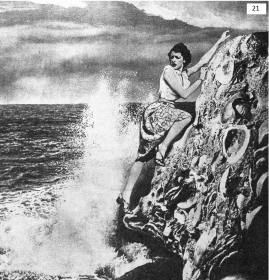 | 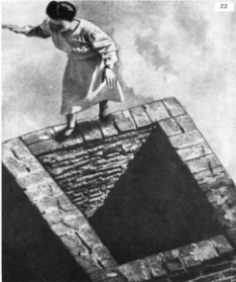 | 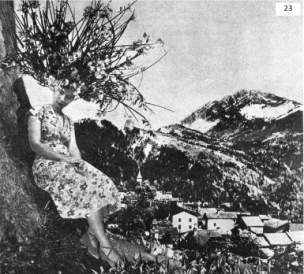 | 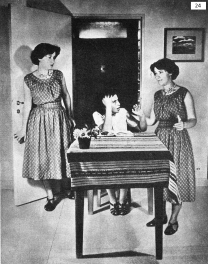 | 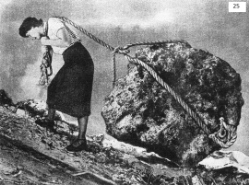 |
| 21  “Idilio_11” | 22  “Idilio_12” | 23  “Idilio_13” | 24  “Idilio_14” | 25  “Idilio_15” |
| 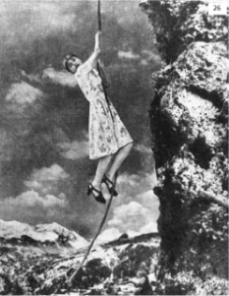 | 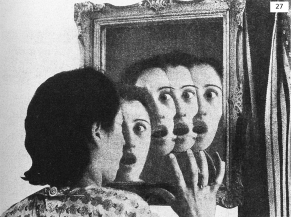 | 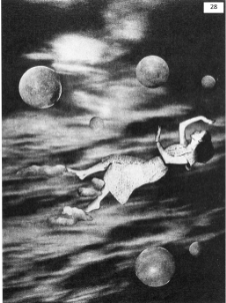 | 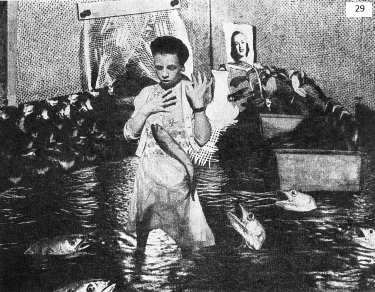 | 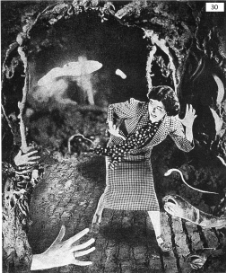 |
| 26  “Idilio_16” | 27  “Idilio_17” | 28  “Idilio_18” | 29  “Idilio_19” | 30  “Idilio_20” |
| 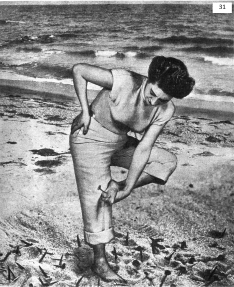 | 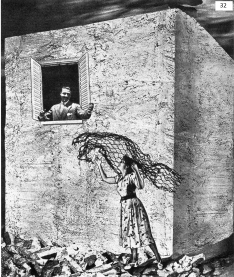 | 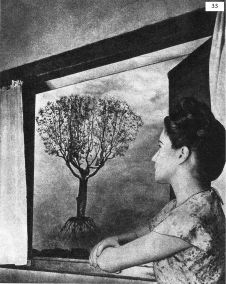 | 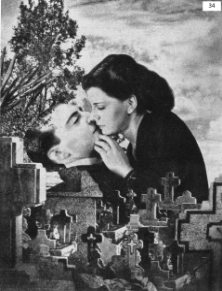 | 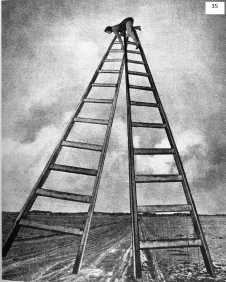 |
| 31  “Idilio_21” | 32  “Idilio_22” | 33  “Idilio_23” | 34  “Idilio_24” | 35  “Idilio_25” |
| 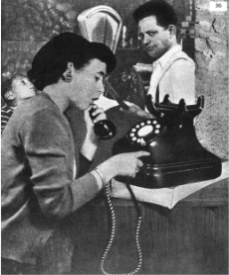 | 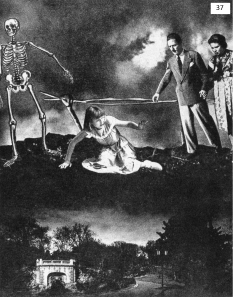 | 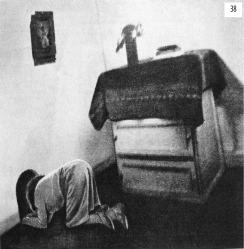 | 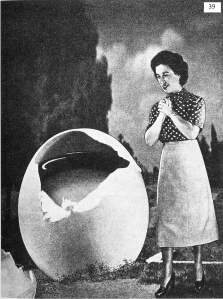 | 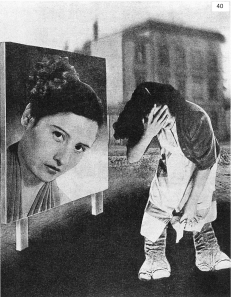 |
| 36  “Idilio_26” | 37  “Idilio_27” | 38  “Idilio_28” | 39  “Idilio_29” | 40  “Idilio_30” |
| 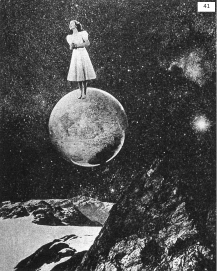 | 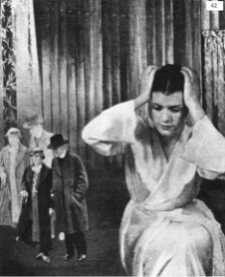 | 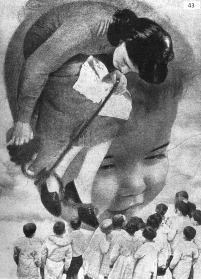 | 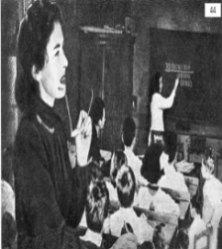 | 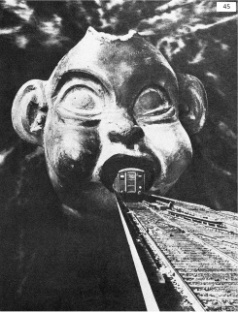 |
| 41  “Idilio_31” | 42  “Idilio_32” | 43  “Idilio_33” | 44  “Idilio_34” | 45  “Idilio_35” |
| 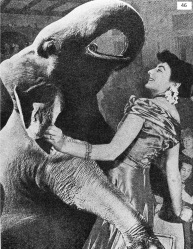 | 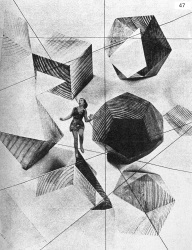 | 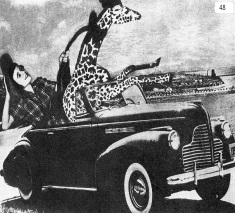 | 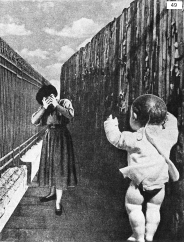 | 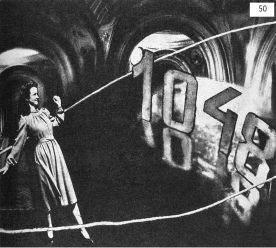 |
| 46  “Idilio_36” | 47  “Idilio_37” | 48  “Idilio_38” | 49  “Idilio_39” | 50  “Idilio_41” |
| 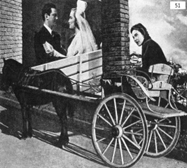 | 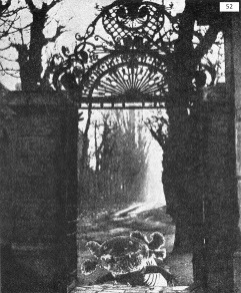 | 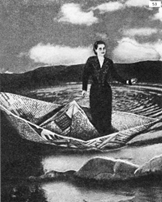 | 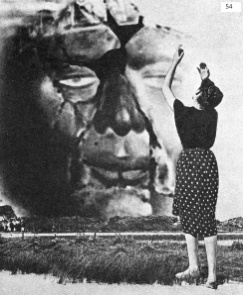 | 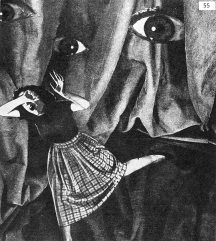 |
| 51  “Idilio_42” | 52  “Idilio_43” | 53  “Idilio_44” | 54  “Idilio_45” | 55  “Idilio_46” |
| 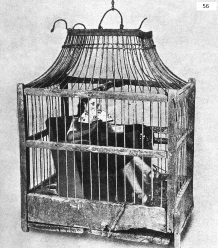 | 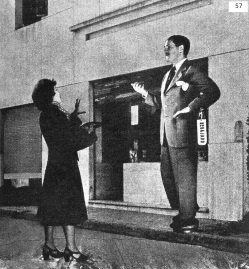 | 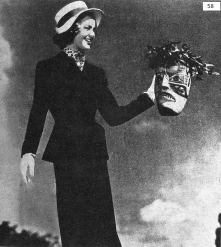 | 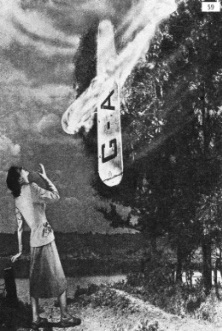 | 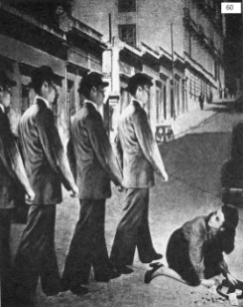 |
| 56  “Idilio_47” | 57  “Idilio_48” | 58  “Idilio_49” | 59  “Idilio_50” | 60  “Idilio_51” |
| 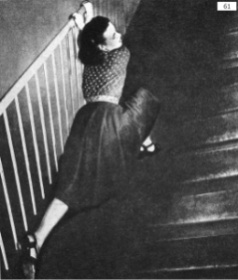 | 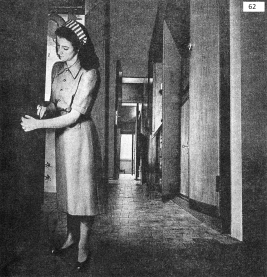 | 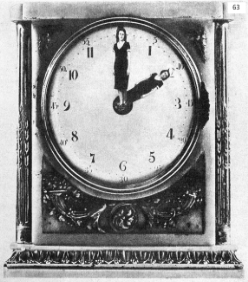 | 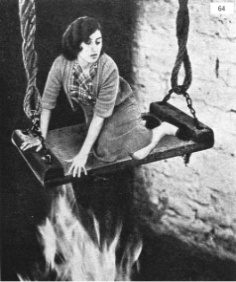 | 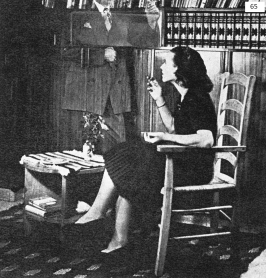 |
| 61  “Idilio_52” | 62  “Idilio_53” | 63  “Idilio_54” | 64  “Idilio_55” | 65  “Idilio_56” |
| 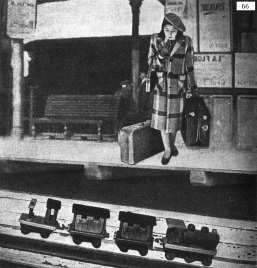 | 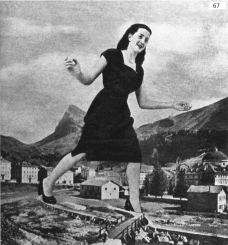 | 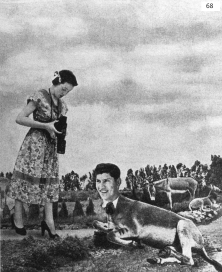 | 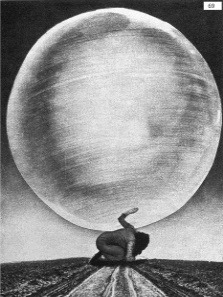 | 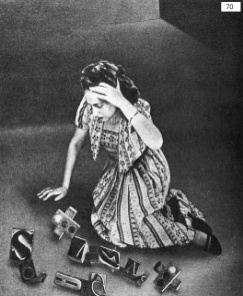 |
| 66  “Idilio_57” | 67  “Idilio_58” | 68  “Idilio_60” | 69  “Idilio_61” | 70  “Idilio_62” |
| 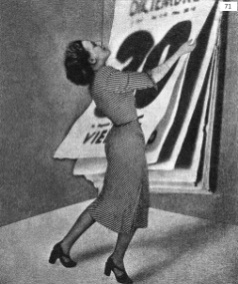 | 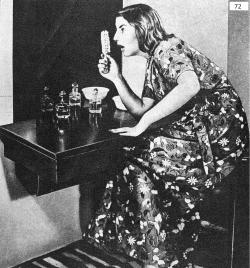 | 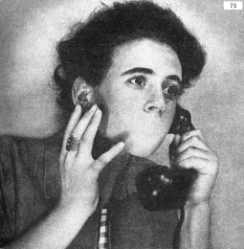 | 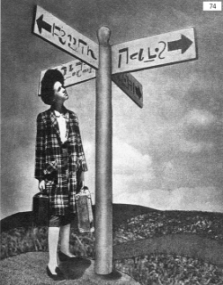 | 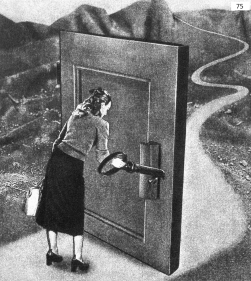 |
| 71  “Idilio_63” | 72  “Idilio_65” | 73  “Idilio_67” | 74  “Idilio_68” | 75  “Idilio_69” |
| 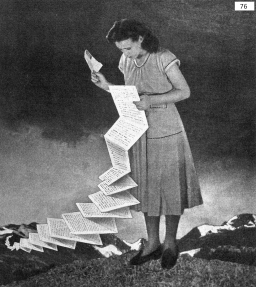 | 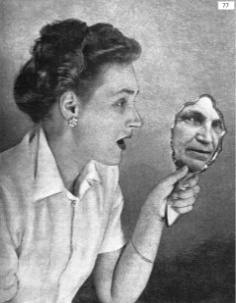 | 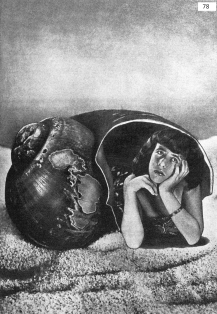 | 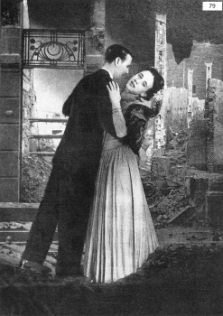 | 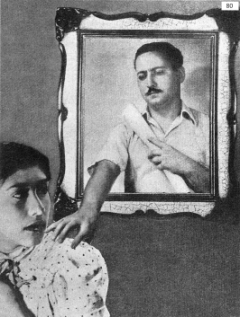 |
| 76  “Idilio_70” | 77  “Idilio_71” | 78  “Idilio_72” | 79  “Idilio_73” | 80  “Idilio_74” |
| 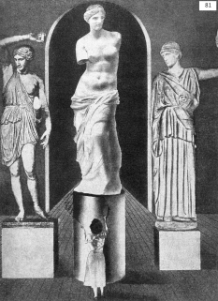 | 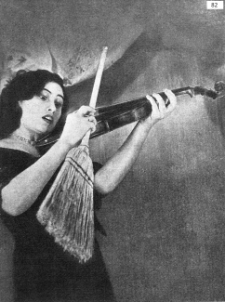 | 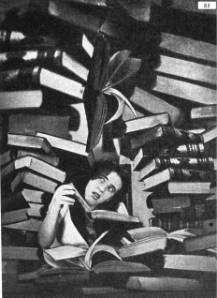 | 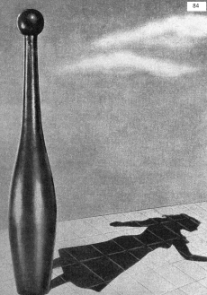 | 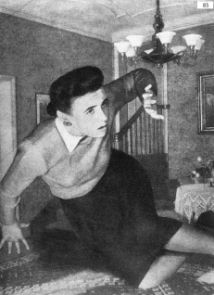 |
| 81  “Idilio_75” | 82  “Idilio_76” | 83  “Idilio_77” | 84  “Idilio_78” | 85  “Idilio_79” |
| 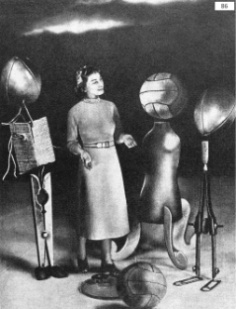 | 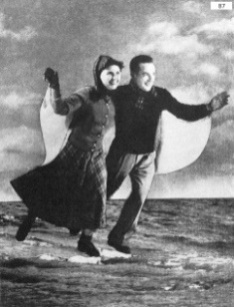 | 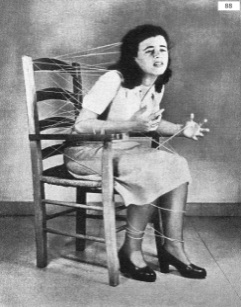 | 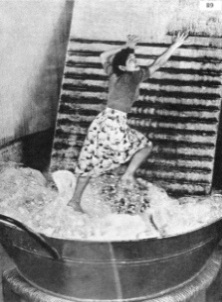 | 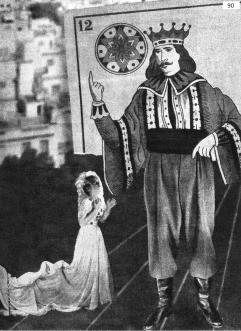 |
| 86  “Idilio_81” | 87  “Idilio_82” | 88  “Idilio_83” | 89  “Idilio_84” | 90  “Idilio_85” |
| 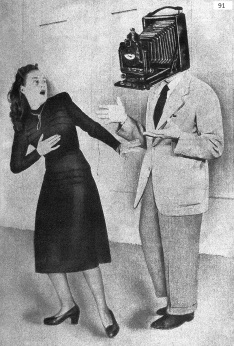 | 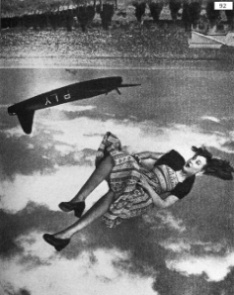 | 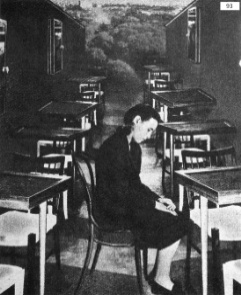 | 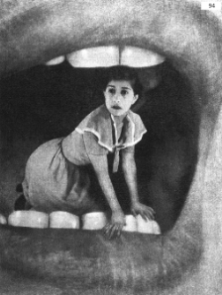 | 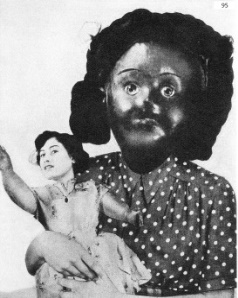 |
| 91  “Idilio_86” | 92  “Idilio_87” | 93  “Idilio_88” | 94  “Idilio_89” | 95  “Idilio_90” |
| 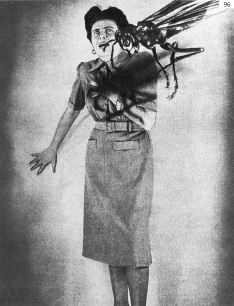 | 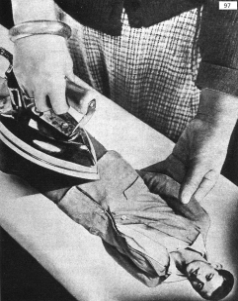 | 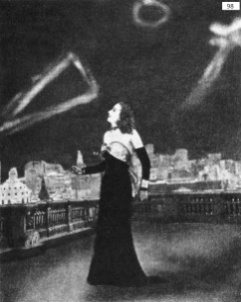 | 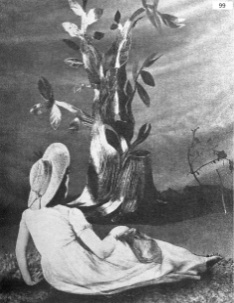 | 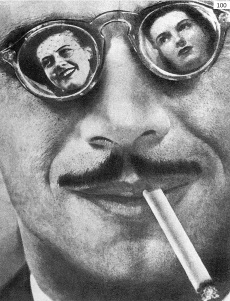 |
| 96  “Idilio_92” | 97  “Idilio_93” | 98  “Idilio_94” | 99  “Idilio_95” | 100  “Idilio_96” |
|  |  |  |  |  |
| 101  “Idilio_97” | 102  “Idilio_98” | 103  “Idilio_99” | 104  “Idilio_100” | 105  “Idilio_102” |
|  |  |  |  |  |
| 106  “Idilio_103” | 107  “Idilio_104” | 108  “Idilio_105” | 109  “Idilio_106” | 110  “Idilio_107” |
|  |  |  |  |  |
| 111  “Idilio_108” | 112  “Idilio_109” | 113  “Idilio_111” | 114  “Idilio_112” | 115  “Idilio_113” |
|  |  |  |  |  |
| 116  “Idilio_114” | 117  “Idilio_115” | 118  “Idilio_116” | 119  “Idilio_117” | 120  “Idilio_118” |
|  |  |  |  |  |
| 121  “Idilio_119” | 122  “Idilio_120” | 123  “Idilio_121” | 124  “Idilio_122” | 125  “Idilio_123” |
|  |  |  |  |  |
| 126  “Idilio_124” | 127  “Idilio_125” | 128  “Idilio_126” | 129  “Idilio_127” | 130  “Idilio_128” |
|  |  |  |  |  |
| 131  “Idilio_129” | 132  “Idilio_130” | 133  “Idilio_132” | 134  “Idilio_133” | 135  “Idilio_134” |
|  |  |  |  |  |
| 136  “Idilio_135” | 137  “Idilio_136” | 138  “Idilio_137” | 139  “Idilio_138” | 140  “Idilio_140” |
