# Supplementary material for: Bizarreness and Emotion Identification in Grete Stern Photomontages: Gender and Age Disparities
Source: Front Psychol. 2017 Mar 22;8:414. doi: 10.3389/fpsyg.2017.00414 (PMC5360721; doi:10.3389/fpsyg.2017.00414)
Supplement: Supplementary file 3 [file Table2.docx]

**2 Table 2**

**Table 2.** The 140 mean and standard deviations (SD) refer to the evaluations of valence, arousal, dominance and bizarreness of Grete Stern photomontages by Young Males (n = 21).

| Name | Slide no. | Valence  Mean (SD) | Arousal  Mean (SD) | Dominance  Mean (SD) | Bizarreness  Mean (SD) |
| --- | --- | --- | --- | --- | --- |
| “Amor sin ilusión” | 1 | 5.05 (2.11) | 5.52 (2.32) | 4.57 (2.69) | 4.29 (2.92) |
| “Artículo eléctricos” | 2 | 4.24 (2.17) | 4.81 (2.38) | 5.95 (2.13) | 5.90 (2.83) |
| “Botella de mar” | 3 | 5.67 (2.46) | 6.48 (2.32) | 4.14 (2.65) | 4.90 (2.70) |
| “Cuerpos Celestes” | 4 | 4.90 (2.72) | 5.81 (2.44) | 5.71 (1.82) | 4.90 (3.05) |
| “El ojo eterno” | 5 | 5.14 (2.80) | 5.38 (2.73) | 5.38 (2.06) | 4.52 (2.96) |
| “En el Andén” | 6 | 5.38 (2.42) | 5.67 (2.37) | 4.95 (2.50) | 4.00 (2.92) |
| “En esta hora” | 7 | 4.00 (2.90) | 6.71 (2.70) | 5.14 (2.24) | 6.90 (2.57) |
| “Los sueños dom…” | 8 | 4.67 (2.13) | 6.81 (2.25) | 5.48 (1.89) | 5.95 (3.01) |
| “Made in England” | 9 | 5.14 (2.24) | 5.48 (1.69) | 4.14 (2.17) | 4.43 (3.17) |
| “No se destiñe agua” | 10 | 4.62 (2.16) | 6.71 (1.85) | 5.05 (2.29) | 6.19 (2.79) |
| “Sirena de mar” | 11 | 4.43 (2.82) | 4.52 (2.18) | 5.67 (2.48) | 4.71 (2.94) |
| “Idilio_1” | 12 | 3.67 (2.20) | 5.95 (2.64) | 5.65 (1.84) | 3.86 (2.52) |
| “Idilio_2” | 13 | 4.81 (2.54) | 6.62 (2.16) | 5.70 (1.63) | 5.85 (2.89) |
| “Idilio_3” | 14 | 5.48 (2.82) | 5.19 (2.52) | 4.90 (2.47) | 4.52 (2.60) |
| “Idilio_4” | 15 | 5.52 (2.68) | 5.45 (2.35) | 5.00 (2.25) | 4.95 (2.38) |
| “Idilio_6” | 16 | 4.48 (2.27) | 4.90 (2.64) | 4.55 (2.52) | 4.90 (2.21) |
| “Idilio_7” | 17 | 4.19 (2.68) | 5.43 (2.69) | 5.50 (2.24) | 6.29 (2.26) |
| “Idilio_8” | 18 | 4.52 (2.89) | 7.14 (2.54) | 5.50 (2.04) | 6.38 (2.65) |
| “Idilio_9” | 19 | 5.38 (2.78) | 6.00 (2.45) | 3.95 (1.93) | 5.33 (2.59) |
| “Idilio_10” | 20 | 4.19 (2.68) | 6.81 (2.18) | 5.15 (2.11) | 5.24 (3.03) |
| “Idilio_11” | 21 | 5.10 (1.92) | 4.85 (2.64) | 4.60 (2.64) | 4.62 (2.42) |
| “Idilio_12” | 22 | 4.48 (2.38) | 5.20 (2.48) | 4.35 (2.37) | 5.05 (2.85) |
| “Idilio_13” | 23 | 5.45 (3.09) | 5.21 (2.30) | 4.68 (2.26) | 5.05 (2.76) |
| “Idilio_14” | 24 | 5.52 (2.71) | 4.85 (2.52) | 4.30 (2.34) | 5.19 (2.68) |
| “Idilio_15” | 25 | 6.00 (2.49) | 4.68 (2.67) | 3.26 (2.23) | 4.75 (2.75) |
| “Idilio_16” | 26 | 5.14 (2.50) | 6.35 (2.48) | 4.75 (2.38) | 3.67 (1.62) |
| “Idilio_17” | 27 | 4.76 (2.83) | 5.20 (2.80) | 5.45 (3.02) | 5.19 (2.82) |
| “Idilio_18” | 28 | 4.71 (2.53) | 5.67 (2.48) | 5.60 (2.33) | 5.57 (2.40) |
| “Idilio_19” | 29 | 5.62 (2.33) | 5.30 (2.45) | 4.65 (3.10) | 4.67 (2.82) |
| “Idilio_20” | 30 | 5.29 (2.87) | 5.15 (2.60) | 4.71 (2.47) | 4.19 (2.79) |
| “Idilio_21” | 31 | 5.48 (2.60) | 4.95 (2.46) | 5.43 (2.34) | 5.48 (2.60) |
| “Idilio_22” | 32 | 3.86 (2.87) | 5.00 (2.61) | 5.55 (1.76) | 6.14 (3.00) |
| “Idilio_23” | 33 | 5.05 (2.99) | 6.65 (2.03) | 5.40 (1.54) | 6.29 (3.08) |
| “Idilio_24” | 34 | 3.81 (2.71) | 5.00 (2.73) | 5.55 (2.37) | 5.43 (3.11) |
| “Idilio_25” | 35 | 5.19 (1.99) | 5.15 (2.56) | 5.86 (2.24) | 5.19 (2.44) |
| “Idilio_26” | 36 | 4.90 (1.61) | 6.05 (2.58) | 6.00 (1.78) | 5.81 (3.28) |
| “Idilio_27” | 37 | 5.95 (2.16) | 4.20 (1.85) | 5.15 (2.35) | 3.86 (2.39) |
| “Idilio_28” | 38 | 5.48 (1.78) | 5.50 (2.14) | 5.15 (2.30) | 4.14 (2.73) |
| “Idilio_29” | 39 | 4.10 (1.89) | 5.67 (2.63) | 5.62 (1.28) | 5.19 (2.52) |
| “Idilio_30” | 40 | 5.95 (1.96) | 5.81 (2.36) | 4.62 (2.16) | 4.10 (2.93) |
| “Idilio_31” | 41 | 3.19 (2.18) | 7.05 (2.38) | 6.24 (2.23) | 5.48 (2.75) |
| “Idilio_32” | 42 | 6.81 (2.09) | 5.00 (2.28) | 4.00 (1.90) | 4.48 (2.44) |
| “Idilio_33” | 43 | 4.86 (2.57) | 6.62 (2.22) | 4.71 (1.93) | 4.43 (2.62) |
| “Idilio_34” | 44 | 5.10 (2.72) | 6.38 (2.50) | 6.29 (2.92) | 7.33 (1.93) |
| “Idilio_35” | 45 | 4.71 (2.63) | 5.19 (2.34) | 5.71 (2.47) | 4.29 (2.51) |
| “Idilio_36” | 46 | 3.71 (2.51) | 4.86 (2.33) | 6.24 (2.21) | 3.90 (2.83) |
| “Idilio_37” | 47 | 3.95 (2.18) | 5.71 (2.35) | 5.29 (2.12) | 5.14 (2.69) |
| “Idilio_38” | 48 | 2.62 (2.16) | 5.85 (2.78) | 5.90 (2.61) | 4.95 (3.01) |
| “Idilio_39” | 49 | 5.48 (2.82) | 6.14 (2.13) | 5.62 (2.20) | 4.71 (2.69) |
| “Idilio_41” | 50 | 3.71 (2.78) | 6.05 (2.50) | 5.86 (2.41) | 5.62 (3.01) |
| “Idilio_42” | 51 | 4.15 (2.18) | 5.19 (2.82) | 6.71 (2.03) | 5.95 (2.87) |
| “Idilio_43” | 52 | 5.76 (2.41) | 5.48 (2.38) | 4.81 (2.27) | 4.19 (1.99) |
| “Idilio_44” | 53 | 4.24 (2.12) | 4.86 (2.65) | 5.71 (1.82) | 5.05 (1.86) |
| “Idilio_45” | 54 | 5.24 (2.79) | 5.67 (2.63) | 4.90 (2.14) | 4.10 (2.45) |
| “Idilio_46” | 55 | 4.67 (2.48) | 4.86 (2.76) | 5.24 (2.76) | 3.57 (2.11) |
| “Idilio_47” | 56 | 5.67 (2.78) | 4.71 (2.78) | 4.71 (2.63) | 4.38 (2.84) |
| “Idilio_48” | 57 | 4.62 (1.86) | 6.43 (2.46) | 6.05 (2.16) | 6.24 (2.23) |
| “Idilio_49” | 58 | 4.14 (2.95) | 5.90 (2.32) | 5.95 (2.50) | 5.19 (2.89) |
| “Idilio_50” | 59 | 4.86 (2.80) | 4.71 (2.99) | 5.52 (2.52) | 4.14 (2.73) |
| “Idilio_51” | 60 | 5.00 (2.53) | 5.05 (2.54) | 5.86 (2.80) | 4.90 (2.49) |
| “Idilio_52” | 61 | 5.14 (2.29) | 5.62 (2.87) | 5.19 (2.36) | 4.95 (2.56) |
| “Idilio_53” | 62 | 5.29 (1.59) | 6.10 (2.28) | 4.86 (1.74) | 5.81 (2.60) |
| “Idilio_54” | 63 | 4.05 (1.99) | 5.62 (2.16) | 5.57 (2.11) | 5.67 (2.71) |
| “Idilio_55” | 64 | 5.80 (2.28) | 4.45 (2.24) | 5.35 (2.37) | 3.85 (2.74) |
| “Idilio_56” | 65 | 4.67 (2.08) | 5.43 (2.42) | 5.52 (1.83) | 5.81 (2.75) |
| “Idilio_57” | 66 | 4.38 (2.65) | 6.76 (2.14) | 5.76 (2.66) | 5.62 (2.80) |
| “Idilio_58” | 67 | 3.33 (2.35) | 4.24 (2.74) | 6.48 (1.83) | 5.71 (2.99) |
| “Idilio_60” | 68 | 5.29 (2.15) | 6.24 (1.95) | 6.14 (2.48) | 5.10 (3.13) |
| “Idilio_61” | 69 | 5.20 (2.12) | 5.20 (2.31) | 4.00 (2.29) | 5.30 (2.83) |
| “Idilio_62” | 70 | 6.10 (2.02) | 5.67 (2.39) | 5.29 (1.74) | 4.76 (2.68) |
| “Idilio_63” | 71 | 5.24 (2.53) | 6.05 (2.71) | 5.05 (2.25) | 5.95 (1.86) |
| “Idilio_65” | 72 | 4.67 (2.31) | 5.90 (2.10) | 5.48 (1.89) | 5.76 (2.93) |
| “Idilio_67” | 73 | 5.80 (1.79) | 5.05 (2.01) | 3.75 (1.97) | 4.05 (1.76) |
| “Idilio_68” | 74 | 4.75 (2.29) | 6.00 (2.55) | 5.15 (2.25) | 5.70 (2.70) |
| “Idilio_69” | 75 | 4.05 (2.94) | 5.52 (2.89) | 5.29 (1.59) | 5.00 (2.68) |
| “Idilio_70” | 76 | 4.60 (2.62) | 6.20 (2.55) | 5.50 (2.24) | 6.35 (2.48) |
| “Idilio_71” | 77 | 4.95 (2.42) | 5.71 (2.22) | 4.62 (2.36) | 4.81 (2.38) |
| “Idilio_72” | 78 | 5.81 (1.99) | 5.90 (2.23) | 5.00 (1.90) | 4.48 (2.75) |
| “Idilio_73” | 79 | 3.62 (2.87) | 5.52 (2.89) | 6.29 (2.19) | 6.10 (2.83) |
| “Idilio_74” | 80 | 5.05 (2.77) | 4.95 (2.42) | 4.24 (2.32) | 4.52 (2.58) |
| “Idilio_75” | 81 | 3.48 (1.78) | 5.71 (1.98) | 5.43 (2.25) | 5.48 (2.60) |
| “Idilio_76” | 82 | 4.10 (2.55) | 5.00 (2.61) | 6.20 (2.44) | 5.38 (2.18) |
| “Idilio_77” | 83 | 4.48 (2.60) | 4.48 (2.64) | 4.67 (2.44) | 5.19 (2.44) |
| “Idilio_78” | 84 | 4.43 (2.11) | 6.52 (2.25) | 5.05 (1.91) | 6.14 (2.63) |
| “Idilio_79” | 85 | 5.48 (2.06) | 5.90 (2.28) | 5.76 (1.92) | 4.90 (2.41) |
| “Idilio_81” | 86 | 5.33 (1.77) | 5.48 (2.52) | 6.05 (1.86) | 5.29 (2.12) |
| “Idilio_82” | 87 | 2.90 (2.26) | 4.90 (2.81) | 5.71 (2.12) | 6.57 (2.77) |
| “Idilio_83” | 88 | 6.10 (2.85) | 4.37 (2.43) | 4.40 (2.91) | 3.65 (2.56) |
| “Idilio_84” | 89 | 5.05 (1.96) | 4.48 (1.94) | 5.19 (1.99) | 4.86 (2.57) |
| “Idilio_85” | 90 | 5.14 (2.54) | 5.00 (2.85) | 5.76 (2.49) | 5.24 (2.57) |
| “Idilio_86” | 91 | 4.95 (2.42) | 5.19 (2.09) | 5.00 (2.37) | 4.14 (2.17) |
| “Idilio_87” | 92 | 4.76 (2.05) | 4.90 (2.70) | 5.35 (1.57) | 4.48 (2.32) |
| “Idilio_88” | 93 | 6.40 (2.68) | 6.05 (1.96) | 4.38 (1.63) | 4.90 (2.64) |
| “Idilio_89” | 94 | 4.76 (1.73) | 4.95 (2.16) | 4.90 (1.84) | 4.86 (2.50) |
| “Idilio_90” | 95 | 4.33 (2.06) | 5.67 (2.13) | 5.14 (1.56) | 4.95 (2.94) |
| “Idilio_92” | 96 | 5.76 (2.14) | 5.05 (2.11) | 4.48 (2.04) | 4.48 (2.52) |
| “Idilio_93” | 97 | 4.81 (2.09) | 5.43 (1.83) | 4.62 (2.44) | 5.24 (2.19) |
| “Idilio_94” | 98 | 4.24 (1.97) | 5.38 (2.18) | 5.24 (1.41) | 5.19 (2.38) |
| “Idilio_95” | 99 | 3.38 (1.94) | 5.90 (2.34) | 5.33 (1.77) | 6.57 (2.29) |
| “Idilio_96” | 100 | 4.05 (2.73) | 5.24 (2.64) | 5.95 (2.25) | 6.24 (2.59) |
| “Idilio_97” | 101 | 3.90 (2.57) | 7.29 (2.28) | 6.48 (2.23) | 6.95 (2.91) |
| “Idilio_98” | 102 | 5.05 (2.06) | 5.86 (2.15) | 4.70 (1.63) | 5.00 (2.19) |
| “Idilio_99” | 103 | 4.00 (2.19) | 4.95 (2.25) | 4.90 (1.77) | 4.76 (2.17) |
| “Idilio_100” | 104 | 3.55 (3.00) | 5.15 (2.30) | 6.26 (2.10) | 5.95 (3.21) |
| “Idilio_102” | 105 | 5.00 (2.19) | 5.48 (2.16) | 4.86 (1.96) | 5.10 (2.26) |
| “Idilio_103” | 106 | 3.43 (2.06) | 5.52 (2.50) | 5.29 (1.59) | 5.81 (2.52) |
| “Idilio_104” | 107 | 3.43 (2.73) | 5.62 (2.54) | 6.19 (2.36) | 5.29 (2.72) |
| “Idilio_105” | 108 | 4.43 (2.36) | 4.90 (2.17) | 5.43 (2.18) | 4.48 (2.62) |
| “Idilio_106” | 109 | 5.62 (2.33) | 5.19 (2.20) | 4.48 (1.54) | 4.62 (2.22) |
| “Idilio_107” | 110 | 5.29 (1.90) | 5.19 (2.16) | 4.81 (1.63) | 4.71 (2.19) |
| “Idilio_108” | 111 | 5.52 (2.64) | 6.05 (2.65) | 6.52 (2.11) | 4.95 (2.73) |
| “Idilio_109” | 112 | 3.71 (1.93) | 5.43 (2.62) | 5.71 (2.55) | 5.90 (2.92) |
| “Idilio_111” | 113 | 5.38 (2.33) | 5.62 (2.29) | 5.29 (1.71) | 5.57 (2.71) |
| “Idilio_112” | 114 | 3.67 (2.20) | 5.90 (2.23) | 5.86 (1.62) | 5.05 (2.73) |
| “Idilio_113” | 115 | 4.10 (2.47) | 5.45 (2.87) | 5.75 (2.07) | 5.80 (2.46) |
| “Idilio_114” | 116 | 3.76 (2.59) | 6.10 (2.64) | 5.86 (1.98) | 6.14 (2.57) |
| “Idilio_115” | 117 | 4.86 (2.24) | 5.52 (2.18) | 5.10 (2.64) | 4.20 (2.86) |
| “Idilio_116” | 118 | 5.38 (2.73) | 5.76 (1.58) | 5.62 (1.94) | 4.90 (2.39) |
| “Idilio_117” | 119 | 4.20 (2.02) | 5.20 (2.33) | 4.95 (2.09) | 5.00 (2.43) |
| “Idilio_118” | 120 | 4.67 (1.88) | 4.71 (2.05) | 5.05 (1.80) | 5.05 (2.36) |
| “Idilio_119” | 121 | 5.57 (2.75) | 6.48 (1.99) | 4.95 (2.25) | 4.62 (2.36) |
| “Idilio_120” | 122 | 4.45 (2.42) | 6.05 (2.33) | 4.80 (2.17) | 6.45 (2.37) |
| “Idilio_121” | 123 | 5.52 (1.12) | 5.62 (1.88) | 4.19 (1.99) | 3.86 (2.17) |
| “Idilio_122” | 124 | 5.24 (2.05) | 5.62 (1.86) | 4.86 (1.49) | 4.43 (2.71) |
| “Idilio_123” | 125 | 4.05 (2.33) | 4.67 (2.48) | 6.00 (2.05) | 5.33 (2.56) |
| “Idilio_124” | 126 | 3.43 (1.80) | 5.19 (2.80) | 5.90 (1.55) | 7.24 (2.28) |
| “Idilio_125” | 127 | 4.43 (2.40) | 6.05 (2.25) | 5.76 (1.61) | 5.05 (2.87) |
| “Idilio_126” | 128 | 4.00 (2.14) | 6.00 (2.24) | 5.43 (1.86) | 6.00 (2.90) |
| “Idilio_127” | 129 | 4.67 (2.03) | 5.81 (2.44) | 5.19 (2.16) | 4.86 (2.76) |
| “Idilio_128” | 130 | 3.29 (2.31) | 5.33 (2.39) | 5.86 (2.15) | 6.52 (2.54) |
| “Idilio_129” | 131 | 5.57 (2.82) | 5.29 (2.10) | 4.86 (2.15) | 4.81 (3.16) |
| “Idilio_130” | 132 | 4.33 (1.80) | 5.24 (2.07) | 5.29 (1.90) | 4.57 (2.91) |
| “Idilio_132” | 133 | 3.81 (2.27) | 5.67 (2.46) | 5.43 (2.16) | 6.33 (2.13) |
| “Idilio_133” | 134 | 5.43 (2.01) | 5.38 (2.16) | 4.57 (1.80) | 4.90 (1.84) |
| “Idilio_134” | 135 | 4.81 (2.42) | 5.48 (2.60) | 5.10 (1.95) | 5.38 (2.75) |
| “Idilio_135” | 136 | 5.00 (2.61) | 5.19 (2.68) | 4.43 (2.48) | 4.33 (2.78) |
| “Idilio_136” | 137 | 3.95 (2.50) | 5.76 (2.39) | 5.38 (2.06) | 5.14 (2.87) |
| “Idilio_137” | 138 | 5.24 (2.10) | 4.67 (2.03) | 5.33 (2.22) | 4.43 (2.48) |
| “Idilio_138” | 139 | 4.33 (1.93) | 5.57 (2.29) | 5.52 (2.27) | 5.19 (2.34) |
| “Idilio_140” | 140 | 3.90 (2.30) | 5.05 (2.33) | 5.52 (1.91) | 4.57 (2.42) |

**Table 3.** The 140 mean and standard deviations (SD) refer to the evaluations of valence, arousal, dominance and bizarreness of Grete Stern photomontages by Young Females (n = 30).

| Name | Slide no. | Valence  Mean (SD) | Arousal  Mean (SD) | Dominance  Mean (SD) | Bizarre  Mean (SD) |
| --- | --- | --- | --- | --- | --- |
| “Amor sin ilusión” | 1 | 4.43 (1.70) | 6.30 (2.39) | 4.41 (2.40) | 3.30 (2.74) |
| “Artículo eléctricos” | 2 | 4.07 (2.20) | 6.30 (2.20) | 5.63 (2.24) | 5.20 (2.93) |
| “Botella de mar” | 3 | 5.07 (2.61) | 6.53 (2.16) | 4.83 (2.45) | 5.40 (3.05) |
| “Cuerpos Celestes” | 4 | 4.80 (2.31) | 5.72 (2.31) | 5.87 (2.10) | 4.43 (2.66) |
| “El ojo eterno” | 5 | 5.93 (2.80) | 5.30 (2.89) | 5.10 (2.41) | 4.31(2.93) |
| “En el Andén” | 6 | 5.55 (2.13) | 4.43 (2.43) | 4.30 (2.42) | 3.27 (2.56) |
| “En esta hora” | 7 | 3.80 (2.91) | 6.67 (2.63) | 5.47 (2.50) | 6.13 (3.35) |
| “Los sueños dom…” | 8 | 4.90 (2.14) | 6.67 (2.47) | 5.83 (2.13) | 4.93 (3.18) |
| “Made in England” | 9 | 6.00 (2.08) | 5.03 (2.27) | 4.73 (2.39) | 4.20 (2.76) |
| “No se destiñe agua” | 10 | 5.57 (2.51) | 5.67 (1.99) | 4.60 (2.59) | 4.90 (2.96) |
| “Sirena de mar” | 11 | 4.66 (2.00) | 5.52 (2.25) | 5.54 (1.90) | 4.17 (2.90) |
| “Idilio_1” | 12 | 4.90 (2.37) | 7.07 (2.17) | 6.10 (2.24) | 5.13 (3.32) |
| “Idilio_2” | 13 | 5.03 (2.43) | 6.79 (2.47) | 4.72 (1.91) | 5.07 (3.17) |
| “Idilio_3” | 14 | 6.13 (2.26) | 4.55 (2.47) | 4.69 (2.32) | 3.14 (2.56) |
| “Idilio_4” | 15 | 7.20 (2.06) | 6.31 (2.35) | 4.07 (2.72) | 4.69 (2.77) |
| “Idilio_6” | 16 | 5.14 (2.33) | 5.03 (2.96) | 4.66 (2.72) | 5.07 (2.95) |
| “Idilio_7” | 17 | 3.41 (2.47) | 7.41 (2.35) | 6.03 (2.11) | 7.13 (2.10) |
| “Idilio_8” | 18 | 3.55 (2.72) | 7.73 (1.86) | 6.38 (2.27) | 6.59 (3.48) |
| “Idilio_9” | 19 | 6.40 (2.04) | 5.86 (2.88) | 4.59 (2.53) | 4.83 (3.07) |
| “Idilio_10” | 20 | 3.62 (2.46) | 6.80 (2.54) | 5.48 (2.31) | 6.59 (2.99) |
| “Idilio_11” | 21 | 4.90 (2.70) | 4.45 (3.11) | 4.62 (2.62) | 5.10 (2.93) |
| “Idilio_12” | 22 | 4.96 (2.56) | 5.36 (2.88) | 5.04 (2.43) | 5.11 (3.15) |
| “Idilio_13” | 23 | 4.03 (2.43) | 6.10 (2.81) | 4.93 (2.64) | 5.34 (3.47) |
| “Idilio_14” | 24 | 5.41 (2.66) | 5.89 (2.77) | 4.19 (2.79) | 5.07 (3.29) |
| “Idilio_15” | 25 | 6.46 (2.50) | 5.30 (2.20) | 4.48 (2.97) | 4.81 (2.77) |
| “Idilio_16” | 26 | 5.24 (2.50) | 5.60 (3.11) | 4.41 (2.32) | 3.79 (2.48) |
| “Idilio_17” | 27 | 4.41 (2.44) | 5.20 (2.91) | 5.31 (2.66) | 5.31 (2.89) |
| “Idilio_18” | 28 | 4.41 (1.80) | 7.10 (2.18) | 4.79 (2.16) | 5.07 (2.80) |
| “Idilio_19” | 29 | 4.97 (2.01) | 5.41 (2.99) | 4.86 (2.28) | 4.13 (2.66) |
| “Idilio_20” | 30 | 5.62 (2.27) | 5.03 (2.34) | 4.28 (2.49) | 3.66 (2.36) |
| “Idilio_21” | 31 | 5.90 (2.73) | 5.23 (2.67) | 5.00 (2.17) | 4.73 (3.31) |
| “Idilio_22” | 32 | 3.70 (2.28) | 5.20 (2.64) | 4.83 (2.26) | 6.87 (2.56) |
| “Idilio_23” | 33 | 5.17 (2.98) | 6.03 (2.65) | 5.28 (2.19) | 5.59 (3.13) |
| “Idilio_24” | 34 | 3.43 (2.70) | 4.70 (3.42) | 5.47 (2.27) | 5.27 (3.52) |
| “Idilio_25” | 35 | 5.14 (2.26) | 4.69 (2.55) | 5.66 (2.51) | 3.79 (3.42) |
| “Idilio_26” | 36 | 4.31 (1.87) | 6.93 (2.23) | 4.79 (2.41) | 5.83 (2.85) |
| “Idilio_27” | 37 | 7.33 (1.83) | 5.30 (2.42) | 3.80 (2.75) | 4.34 (3.12) |
| “Idilio_28” | 38 | 5.70 (2.51) | 6.00 (2.56) | 4.47 (2.52) | 4.03 (2.93) |
| “Idilio_29” | 39 | 3.30 (2.28) | 5.20 (2.99) | 5.33 (2.17) | 6.07 (3.14) |
| “Idilio_30” | 40 | 6.67 (2.41) | 5.23 (2.51) | 5.67 (2.06) | 4.07 (3.10) |
| “Idilio_31” | 41 | 3.57 (2.49) | 6.53 (2.66) | 5.87 (1.93) | 5.40 (3.25) |
| “Idilio_32” | 42 | 6.27 (2.49) | 6.43 (2.25) | 4.10 (2.68) | 4.63 (2.88) |
| “Idilio_33” | 43 | 4.27 (2.85) | 6.60 (2.54) | 5.00 (2.10) | 4.60 (3.46) |
| “Idilio_34” | 44 | 3.90 (1.71) | 6.60 (2.54) | 5.97 (2.39) | 6.50 (2.50) |
| “Idilio_35” | 45 | 6.23 (2.34) | 4.93 (2.26) | 3.90 (2.58) | 2.87 **(**2.87) |
| “Idilio_36” | 46 | 3.30 (2.05) | 6.13 (2.71) | 6.00(2.56) | 5.37 (3.33) |
| “Idilio_37” | 47 | 4.30 (2.48) | 5.23 (3.01) | 5.67 (2.12) | 5.13 (3.01) |
| “Idilio_38” | 48 | 2.60 (2.19) | 5.23 (3.08) | 6.33 (2.70) | 4.67 (3.15) |
| “Idilio_39” | 49 | 4.60 (3.21) | 5.27 (2.15) | 5.40 (2.31) | 4.00 (3.10) |
| “Idilio_41” | 50 | 3.87 (2.56) | 5.13 (2.40) | 4.90 (2.20) | 5.80 (2.66) |
| “Idilio_42” | 51 | 4.60 (2.90) | 6.43 (2.85) | 5.20 (2.48) | 5.50 (2.93) |
| “Idilio_43” | 52 | 5.00 (2.10) | 5.57 (2.22) | 5.13 (1.96) | 4.47 (2.67) |
| “Idilio_44” | 53 | 4.20 (2.33) | 5.97 (2.63) | 5.70 (2.31) | 5.70 (2.56) |
| “Idilio_45” | 54 | 5.37 (2.63) | 4.93 (2.75) | 5.23 (2.22) | 3.67 (3.03) |
| “Idilio_46” | 55 | 6.70 (2.37) | 4.86 (2.61) | 3.97 (2.41) | 3.20 (2.75) |
| “Idilio_47” | 56 | 6.23 (1.89) | 5.47 (2.45) | 4.00 (2.55) | 4.30 (2.45) |
| “Idilio_48” | 57 | 4.30 (2.34) | 7.23 (2.13) | 4.77 (2.51) | 6.27 (2.86) |
| “Idilio_49” | 58 | 3.70 (1.76) | 6.73 (2.21) | 5.53 (2.03) | 6.13 (2.56) |
| “Idilio_50” | 59 | 5.83 (2.38) | 4.93 (2.38) | 4.83 (2.00) | 3.73 (2.49) |
| “Idilio_51” | 60 | 7.23 (2.19) | 4.50 (2.42) | 4.00 (2.60) | 3.53 (2.83) |
| “Idilio_52” | 61 | 4.77 (2.40) | 5.37 (2.44) | 5.57 (2.25) | 5.47 (3.13) |
| “Idilio_53” | 62 | 5.40 (1.69) | 6.40 (1.90) | 5.17 (1.66) | 6.27 (2.80) |
| “Idilio_54” | 63 | 4.33 (2.37) | 5.90 (2.88) | 5.10 (2.52) | 5.13 (3.05) |
| “Idilio_55” | 64 | 6.07 (2.02) | 4.83 (2.68) | 3.77 (2.22) | 3.30 (2.48) |
| “Idilio_56” | 65 | 4.55 (2.10) | 6.24 (2.23) | 5.48 (2.16) | 6.17 (2.85) |
| “Idilio_57” | 66 | 5.00 (2.73) | 5.93 (2.77) | 5.07 (2.49) | 5.47 (3.09) |
| “Idilio_58” | 67 | 2.90 (2.55) | 5.07 (3.46) | 6.93 (2.26) | 6.23 (2.87) |
| “Idilio_60” | 68 | 3.67 (2.54) | 5.43 (3.06) | 6.50 (2.66) | 4.87 (3.28) |
| “Idilio_61” | 69 | 5.07 (2.49) | 5.07 (2.49) | 3.93 (2.66) | 3.87 (3.15) |
| “Idilio_62” | 70 | 6.34 (1.82) | 6.28 (2.05) | 4.86 (1.85) | 4.66 (2.73) |
| “Idilio_63” | 71 | 3.70 (2.48) | 6.53 (2.66) | 5.53 (2.34) | 6.20 (3.35) |
| “Idilio_65” | 72 | 4.53 (2.15) | 6.17 (2.88) | 5.17 (2.26) | 5.37 (3.26) |
| “Idilio_67” | 73 | 6.77 (1.81) | 5.20 (2.37) | 2.97 (2.11) | 2.90 (2.50) |
| “Idilio_68” | 74 | 5.33 (2.35) | 6.03 (2.50) | 4.30 (2.02) | 3.80 (2.91) |
| “Idilio_69” | 75 | 4.67 (2.23) | 5.43 (2.51) | 5.57 (2.75) | 5.03 (3.35) |
| “Idilio_70” | 76 | 5.17 (2.17) | 5.90 (2.47) | 5.07 (1.78) | 4.57 (2.80) |
| “Idilio_71” | 77 | 5.73 (2.60) | 4.73 (2.50) | 5.07 (2.60) | 3.67 (3.03) |
| “Idilio_72” | 78 | 5.60 (2.18) | 6.60 (2.19) | 4.73 (2.39) | 5.00 (3.02) |
| “Idilio_73” | 79 | 3.57 (2.97) | 5.20 (3.25) | 4.33 (2.01) | 7.37 (2.58) |
| “Idilio_74” | 80 | 5.90 (2.38) | 5.23 (2.28) | 4.73 (2.16) | 3.67 (3.08) |
| “Idilio_75” | 81 | 4.00 (1.72) | 6.93 (2.65) | 5.53 (2.16) | 6.53 (3.05) |
| “Idilio_76” | 82 | 3.63 (2.33) | 5.97 (2.98) | 4.90 (1.90) | 5.53 (3.10) |
| “Idilio_77” | 83 | 5.13 (2.34) | 5.00 (2.63) | 4.83 (2.53) | 5.23 (3.06) |
| “Idilio_78” | 84 | 4.57 (1.74) | 6.66 (2.33) | 4.43 (2.06) | 4.07 (2.73) |
| “Idilio_79” | 85 | 5.17 (2.17) | 4.77 (2.25) | 4.63 (2.68) | 4.87 (2.87) |
| “Idilio_81” | 86 | 4.00 (2.27) | 6.17 (2.55) | 5.83 (2.17) | 4.53 (2.81) |
| “Idilio_82” | 87 | 2.23 (1.96) | 4.83 (3.17) | 5.20 (2.12) | 6.93 (2.70) |
| “Idilio_83” | 88 | 7.07 (2.38) | 4.37 (2.20) | 2.60 (1.94) | 4.03 (3.18) |
| “Idilio_84” | 89 | 6.67 (1.97) | 5.00 (2.46) | 4.17 (2.76) | 3.90 (2.76) |
| “Idilio_85” | 90 | 5.50 (2.24) | 5.40 (1.92) | 4.73 (2.86) | 4.67 (2.97) |
| “Idilio_86” | 91 | 5.03 (1.63) | 4.93 (2.30) | 5.13 (2.30) | 3.33 (3.07) |
| “Idilio_87” | 92 | 4.97 (2.50) | 4.20 (2.61) | 4.27 (2.55) | 4.53 (3.18) |
| “Idilio_88” | 93 | 7.67 (1.69) | 6.33 (2.75) | 4.20 (1.86) | 3.13 (2.57) |
| “Idilio_89” | 94 | 5.73 (1.70) | 4.73 (2.66) | 4.70 (2.42) | 3.77 (2.78) |
| “Idilio_90” | 95 | 4.27 (2.07) | 5.67 (2.31) | 5.21 (2.35) | 4.17 (3.05) |
| “Idilio_92” | 96 | 6.30 (2.02) | 4.80 (2.75) | 3.87 (2.76) | 3.77 (2.92) |
| “Idilio_93” | 97 | 4.50 (1.98) | 6.13 (2.61) | 6.20 (2.91) | 5.00 (2.92) |
| “Idilio_94” | 98 | 3.40 (1.77) | 5.60 (3.11) | 5.87 (2.15) | 5.87 (3.05) |
| “Idilio_95” | 99 | 3.60 (2.04) | 6.47 (2.78) | 5.60 (1.83) | 6.47 (2.57) |
| “Idilio_96” | 100 | 3.67 (2.84) | 4.67 (2.88) | 5.67 (2.70) | 5.93 (3.18) |
| “Idilio_97” | 101 | 2.87 (2.34) | 6.87 (2.83) | 6.07 (2.33) | 7.07 (2.80) |
| “Idilio_98” | 102 | 4.87 (2.34) | 5.93 (2.45) | 4.80 (2.31) | 4.33 (3.28) |
| “Idilio_99” | 103 | 4.33 (1.92) | 4.93 (2.32) | 4.40 (1.98) | 3.87 (3.14) |
| “Idilio_100” | 104 | 2.87 (2.52) | 5.07 (3.22) | 5.37 (1.79) | 7.30 (2.51) |
| “Idilio_102” | 105 | 4.60 (1.69) | 6.28 (2.05) | 4.80 (2.25) | 4.53 (2.45) |
| “Idilio_103” | 106 | 3.47 (2.15) | 5.67 (2.64) | 5.66 (2.18) | 5.67 (2.64) |
| “Idilio_104” | 107 | 2.27 (1.70) | 5.60 (2.63) | 5.27 (2.55) | 6.30 (2.45) |
| “Idilio_105” | 108 | 5.47 (2.15) | 5.60 (2.24) | 3.73 (2.20) | 3.97 (2.93) |
| “Idilio_106” | 109 | 6.00 (2.85) | 5.43 (2.06) | 4.77 (2.13) | 4.76 (3.07) |
| “Idilio_107” | 110 | 6.33 (1.77) | 4.80 (2.25) | 3.97 (2.30) | 4.23 (2.94) |
| “Idilio_108” | 111 | 6.21 (2.23) | 6.27 (2.38) | 5.03 (2.36) | 4.77 (2.99) |
| “Idilio_109” | 112 | 3.33 (2.40) | 5.10 (3.13) | 5.73 (2.55) | 5.67 (3.03) |
| “Idilio_111” | 113 | 5.63 (2.27) | 6.23 (2.37) | 5.37 (2.11) | 4.83 (3.09) |
| “Idilio_112” | 114 | 5.10 (2.11) | 5.60 (2.30) | 5.33 (1.95) | 5.27 (2.77) |
| “Idilio_113” | 115 | 2.63 (2.01) | 5.40 (2.99) | 6.03 (2.71) | 6.73 (2.50) |
| “Idilio_114” | 116 | 2.27 (2.26) | 6.40 (2.98) | 5.77 (1.96) | 6.40 (3.01) |
| “Idilio_115” | 117 | 5.63 (2.50) | 5.13 (2.67) | 4.53 (2.15) | 3.13 (2.67) |
| “Idilio_116” | 118 | 5.90 (2.71) | 5.87 (2.71) | 4.63 (1.90) | 4.52 (3.37) |
| “Idilio_117” | 119 | 4.73 (3.02) | 6.07 (2.96) | 4.66 (2.39) | 5.27 (3.18) |
| “Idilio_118” | 120 | 5.60 (2.30) | 4.30 (2.79) | 4.67 (2.73) | 4.13 (3.18) |
| “Idilio_119” | 121 | 6.33 (2.73) | 6.97 (2.24) | 5.40 (2.27) | 5.00 (3.30) |
| “Idilio_120” | 122 | 4.37 (2.20) | 5.27 (3.05) | 5.97 (2.24) | 5.07 (2.95) |
| “Idilio_121” | 123 | 5.97 (2.14) | 5.30 (2.67) | 4.21 (2.13) | 3.07 (2.80) |
| “Idilio_122” | 124 | 5.53 (2.22) | 5.48 (2.49) | 4.43 (2.06) | 4.23 (2.70) |
| “Idilio_123” | 125 | 4.43 (2.19) | 5.53 (2.67) | 5.27 (2.21) | 6.67 (2.58) |
| “Idilio_124” | 126 | 3.40 (1.71) | 5.73 (2.85) | 5.87 (2.34) | 7.83 (1.70) |
| “Idilio_125” | 127 | 4.03 (1.87) | 5.27 (3.00) | 4.34 (1.93) | 5.70 (3.19) |
| “Idilio_126” | 128 | 3.47 (2.19) | 6.67 (2.63) | 5.40 (2.11) | 5.97 (2.98) |
| “Idilio_127” | 129 | 5.03 (1.97) | 5.97 (2.53) | 5.43 (2.11) | 4.10 (3.02) |
| “Idilio_128” | 130 | 2.77 (2.25) | 5.47 (2.96) | 5.83 (1.82) | 7.27 (2.39) |
| “Idilio_129” | 131 | 5.27 (2.82) | 5.87 (2.39) | 5.03 (2.41) | 5.13 (3.32) |
| “Idilio_130” | 132 | 4.70 (1.47) | 6.37 (2.17) | 4.97 (1.97) | 4.77 (2.99) |
| “Idilio_132” | 133 | 3.40 (2.06) | 5.40 (2.94) | 5.17 (2.04) | 6.40 (2.84) |
| “Idilio_133” | 134 | 5.77 (2.51) | 5.60 (2.30) | 4.73 (2.61) | 4.07 (2.66) |
| “Idilio_134” | 135 | 5.10 (1.54) | 6.40 (2.53) | 5.03 (1.94) | 5.83 (2.88) |
| “Idilio_135” | 136 | 5.34 (2.78) | 5.10 (2.93) | 5.07 (2.55) | 4.13 (3.22) |
| “Idilio_136” | 137 | 3.23 (2.22) | 6.00 (2.77) | 6.10 (2.20) | 6.00 (2.91) |
| “Idilio_137” | 138 | 5.90 (2.01) | 5.60 (2.18) | 4.43 (2.16) | 4.10 (2.68) |
| “Idilio_138” | 139 | 5.10 (2.38) | 6.43 (2.37) | 4.50 (2.05) | 5.70 (2.72) |
| “Idilio_140” | 140 | 4.93 (2.85) | 5.80 (2.95) | 5.03 (2.20) | 5.90 (2.98) |
