# Supplementary material for: Bizarreness and Emotion Identification in Grete Stern Photomontages: Gender and Age Disparities
Source: Front Psychol. 2017 Mar 22;8:414. doi: 10.3389/fpsyg.2017.00414 (PMC5360721; doi:10.3389/fpsyg.2017.00414)
Supplement: Supplementary file 5 [file Table4.docx]

**Table 4.** Evaluations of valence, arousal, dominance and bizarreness of Grete Stern photomontages by Old Males (n = 18).

| Name | Slide no. | Valence  Mean (SD) | Arousal  Mean (SD) | Dominance  Mean (SD) | Bizarreness  Mean (SD) |
| --- | --- | --- | --- | --- | --- |
| “Amor sin ilusión” | 1 | 4.39 (2.85) | 6.33 (2.66) | 5.72 (2.42) | 5.00 (3.29) |
| “En el Andén” | 2 | 4.83 (2.96) | 5.94 (2.75) | 5.61 (2.40) | 6.61 (2.89) |
| “En esta Hora” | 3 | 3.61 (2.97) | 6.39 (2.20) | 6.17 (2.43) | 5.50 (3.03) |
| “Idilio_3” | 4 | 7.06 (2.13) | 4.83 (2.92) | 4.61 (2.45) | 4.28 (3.03) |
| “Idilio_7” | 5 | 2.50 (1.34) | 6.67 (2.38) | 4.89 (2.45) | 5.94 (2.80) |
| “Idilio_8” | 6 | 3.94 (2.13) | 6.39 (2.68) | 4.94 (2.80) | 5.83 (2.79) |
| “Idilio_16” | 7 | 5.28 (2.70) | 5.00 (2.93) | 4.67 (2.17) | 4.17 (2.53) |
| “Idilio_20” | 8 | 5.28 (3.03) | 5.06 (2.58) | 3.89 (2.52) | 5.00 (2.97) |
| “Idilio_23” | 9 | 3.67 (2.35) | 6.17 (2.62) | 6.17 (2.71) | 5.50 (2.83) |
| “Idilio_25” | 10 | 5.06(3.23) | 5.28 (3.32) | 5.17 (2.38) | 5.39 (3.35) |
