# Supplementary material for: Bizarreness and Emotion Identification in Grete Stern Photomontages: Gender and Age Disparities
Source: Front Psychol. 2017 Mar 22;8:414. doi: 10.3389/fpsyg.2017.00414 (PMC5360721; doi:10.3389/fpsyg.2017.00414)
Supplement: Supplementary file 6 [file Table5.docx]

**Table 5.** Evaluations of valence, arousal, dominance and bizarreness of Grete Stern photomontages by Old Females (n = 28).

| Name | Slide no. | Valence  Mean (SD) | Arousal  Mean (SD) | Dominance  Mean (SD) | Bizarreness  Mean (SD) |
| --- | --- | --- | --- | --- | --- |
| “Amor sin ilusión” | 1 | 4.89 (2.75) | 5.64 (2.44) | 4.89 (2.44) | 4.30 (3.39) |
| “En el Andén” | 2 | 5.29 (2.42) | 5.25 (2.61) | 4.79 (2.45) | 3.64 (2.88) |
| “En esta Hora” | 3 | 4.21 (2.57) | 6.21 (2.69) | 4.86 (2.24) | 6.21 (2.95) |
| “Idilio_3” | 4 | 6.57 (2.79) | 4.29 (2.26) | 4.36 (2.18) | 4.43 (2.92) |
| “Idilio_7” | 5 | 2.71 (2.54) | 6.36 (2.11) | 5.43 (2.63) | 6.36 (2.93) |
| “Idilio_8” | 6 | 2.69 (2.51) | 7.23 (2.55) | 5.62 (2.10) | 5.69 (2.99) |
| “Idilio_16” | 7 | 5.35 (3.19) | 4.08 (2.95) | 4.96 (2.81) | 5.27 (3.34) |
| “Idilio_20” | 8 | 6.04 (2.86) | 4.46 (3.08) | 3.69 (2.40) | 3.24 (2.85) |
| “Idilio_23” | 9 | 3.65 (2.61) | 7.15 (2.82) | 5.62 (2.45) | 6.62 (3.20) |
| “Idilio_25” | 10 | 4.65(2.90) | 4.62 (3.05) | 4.81 (2.43) | 4.62 (3.35) |
